# Immediate Transcriptional Response to a Temperature Pulse under a Fluctuating Thermal Regime

**DOI:** 10.1093/icb/icz096

**Published:** 2019-06-07

**Authors:** Dacotah Melicher, Alex S Torson, Tanner J Anderson, George D Yocum, Joseph P Rinehart, Julia H Bowsher

**Affiliations:** 1 U.S. Department of Agriculture/Agricultural Research Service, Bioscience Research Laboratory, Edward T. Schafer Agricultural Research Center, 1616 Albrecht Boulevard, Fargo, ND 58102, USA; 2 Department of Biology, University of Western Ontario, London, ON N6A 5B7, Canada; 3 Department of Biological Sciences, North Dakota State University, 1340 Bolley Drive, 218 Stevens Hall, Fargo, ND 58102, USA; 4 Department of Anthropology, University of Oregon, 1585 E 13th Ave., Eugene, OR 97403, USA

## Abstract

The response of ectotherms to temperature stress is complex, non-linear, and is influenced by life stage and previous thermal exposure. Mortality is higher under constant low temperatures than under a fluctuating thermal regime (FTR) that maintains the same low temperature but adds a brief, daily pulse of increased temperature. Long term exposure to FTR has been shown to increase transcription of genes involved in oxidative stress, immune function, and metabolic pathways, which may aid in recovery from chill injury and oxidative damage. Previous research suggests the transcriptional response that protects against sub-lethal damage occurs rapidly under exposure to fluctuating temperatures. However, existing studies have only examined gene expression after a week or over many months. Here we characterize gene expression during a single temperature cycle under FTR. Development of pupating alfalfa leafcutting bees (*Megachile rotundata)* was interrupted at the red-eye stage and were transferred to 6°C with a 1-h pulse to 20°C and returned to 6°C. RNA was collected before, during, and after the temperature pulse and compared to pupae maintained at a static 6°C. The warm pulse is sufficient to cause expression of transcripts that repair cell membrane damage, modify membrane composition, produce antifreeze proteins, restore ion homeostasis, and respond to oxidative stress. This pattern of expression indicates that even brief exposure to warm temperatures has significant protective effects on insects exposed to stressful cold temperatures that persist beyond the warm pulse. *Megachile rotundata’s* sensitivity to temperature fluctuations indicates that short exposures to temperature changes affect development and physiology. Genes associated with developmental patterning are expressed after the warm pulse, suggesting that 1 h at 20°C was enough to resume development in the pupae. The greatest difference in gene expression occurred between pupae collected after the warm pulse and at constant low temperatures. Although both were collected at the same time and temperature, the transcriptional response to one FTR cycle included multiple transcripts previously identified under long-term FTR exposure associated with recovery from chill injury, indicating that the effects of FTR occur rapidly and are persistent.

## Introduction

Insects respond to temperature stress in a way that is non-linear and is influenced by life stage and previous thermal exposure (Sinclair et al. 2016). Cold tolerance often varies across life stages (Jensen et al. 2007). Exposure to cold stress during life stages that are not physiologically prepared for cold experience damage, which can cause acute mortality or can accumulate resulting in sub-lethal effects on fitness (Whitfield and Richards 1992; Yocum et al. 1994; Renault et al. 2004; Yocum et al. 2006; Bale and Hayward 2010; Bennett et al. 2013; Hayward et al. 2014). Depending on severity, cold temperatures cause freezing injury, direct chilling injury, or indirect chilling injury (Denlinger and Lee 2010). Indirect chill injury is an accumulation of damage caused by extended cold exposure, which harms cell membranes, disrupts ion balance, and causes oxidative damage (Rojas and Leopold 1996; Koštál et al. 2004, 2006; Lalouette et al. 2011). These physiological effects are often deleterious to the insect’s performance and can decrease survival (Whitfield and Richards 1992; Yocum et al. 1994, 2006; Renault et al. 2004; Bale and Hayward 2010; Colinet et al. 2011; Bennett et al. 2013). While indirect chill injury causes damage to cells and increased mortality, periodically increasing temperatures during cold exposure increases survival (Rinehart et al. 2011, 2013). These temperature fluctuations are frequently referred to as fluctuating thermal regimes (FTRs) (Koštál et al. 2007; Rinehart et al. 2016). Experiments with FTR have demonstrated a benefit in many temperature contexts and across a broad range of insect species (Chen and Denlinger 1992; Renault et al. 2004; Colinet et al. 2006, 2015) and life stages (Renault et al. 2004; Koštál et al. 2007; Torson et al. 2015, 2017). Variation in experimental design across studies makes it difficult to form broad conclusions about the mechanistic basis of the protective effects (Colinet et al. 2015), but recovering ion balance (Koštál et al. 2007) and metabolic homeostasis (Colinet et al. 2016) appear to be conserved benefits of fluctuating temperatures. How these responses are transcriptionally regulated is unclear (Colinet et al. 2018).

*Megachile rotundata*, the alfalfa leafcutting bee, is an extensively studied system for investigating indirect chill injury with a well-characterized, beneficial response to FTR (Rinehart et al. 2016). Bees are exposed to cold during two life stages, overwintering pre-pupae and developing pupae. Adult *M. rotundata* emerge in the early summer and females construct brood cells soon after emergence (Pitts-Singer and Cane 2011). The larvae develop until the fifth instar and then enter diapause for the winter (Pitts-Singer and Cane 2011). Agricultural producers of *M. rotundata* store diapausing pre-pupae in constant temperature (CT) cold storage (Pitts-Singer and Cane 2011), which causes indirect chill injury over extended storage (Rinehart et al. 2013). When adults are needed for spring pollination, pre-pupae are transferred to 29°C, which initiates pupation and adult emergence in ∼20 days. If poor weather delays alfalfa bloom, managers return pupae to CT cold storage, which may cause indirect chill injury and sub-lethal effects on adult performance (Rinehart et al. 2011; Bennett et al. 2015). Storage under FTR improves survival and reduces sub-lethal effects in both overwintering and cold-stored pupae (Bennett et al. 2013, 2015; Rinehart et al. 2013, 2016).

Analysis of gene expression during exposure to fluctuating temperatures has supported the mechanisms identified through physiological experiments. Overwintering *M. rotundata* prepupae exposed to fluctuating temperatures over a 7-month period caused up-regulation of transcripts involved in metabolic activity, ion homeostasis, immune response, and response to oxidative stress (Torson et al. 2015). Developing pupae exposed to the same temperatures showed up-regulation of transcripts involved in similar processes, but the specific transcripts involved were different from the overwintering stage (Torson et al. 2017). These experiments demonstrate that the transcriptomic response to FTR is rapid, with changes to gene expression established after a week of exposure (Torson et al. 2017), and have long term effects on survival (Torson et al. 2015). While these studies reveal possible mechanisms for repair of and protection against chill injury under FTR, short-term transcriptional effects could confirm the patterns observed in prior studies while also establishing timing of the response. Furthermore, previous studies have only investigated gene expression during the cold phase of FTR (Torson et al. 2015, 2017), while cellular mechanisms are likely to be up-regulated during the warm pulse.

The goal of this study was to capture the transcriptional response to FTR in developing pupae by measuring gene expression prior to, during, and after a cycle of a fluctuating thermal regime. *Megachile rotundata* pupae were allowed to develop to the red-eye stage before pupae development was interrupted by storage under FTR and CT treatments. The transcriptional response was compared before, during, and after the warm pulse to pupae left at CT 6°C. We identified specific pathways involved in the prevention of indirect chill injury and confirmed that exposure to a single warm pulse can have a significant and lasting effect on developing pupae. In addition, we found transcripts that were identified in previous studies with longer treatment durations showed an immediate transcriptional response after one warm pulse.

## Materials and methods

### Insects and temperature protocols

**Fig. 1:**
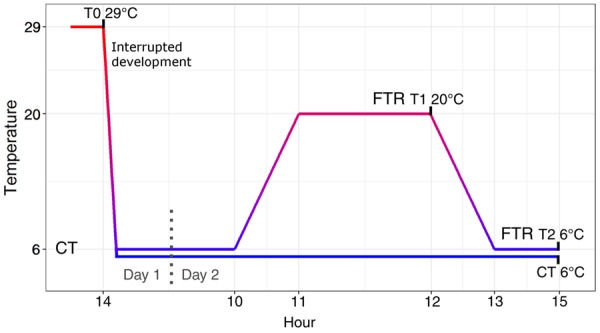
mRNA sampling strategy for differential gene expression analysis during FTR pulse. Pupae were allowed to develop at 29°C until the red-eye pigmentation stage. mRNA was extracted from pupae prior to treatment (T0), during (T1), and after the warm pulse (T2). Pupae at 29°C were collected immediately prior to the temperature treatments (T0). Pupae were transferred to FTR and CT treatments. Pupae were collected at the end of a 1 h 20°C warm pulse (T1) and 2 h after returning to 6°C (T2). The 6°C CT treatment was also sampled at the T2 time point.

Alfalfa leafcutting bees (*M. rotundata*) were obtained from JWM Leafcutters, Inc. (Nampa, ID). Pre-pupae were stored at 6°C under darkness to maintain diapause until the start of the experiment. Prior to the experiment, pre-pupae were transferred to a 29°C incubator to initiate development in cell culture plates. Three replicate plates were used for each sampled time point. Pre-pupae were placed in two reporter plates to monitor development. After 50% of the bees in the reporter plates had developed to the red-eye stage (14–15 days of development, 5 days prior to adult emergence), bees were transferred to FTR and CT treatments to interrupt development. The CT treated bees were stored at a CT of 6°C under darkness. The FTR treatment was exposed to 6°C with a warm pulse of 20°C which occurred between 11:00 and 12:00 with a 1 h ramp to 20°C, 1 h incubation at 20°C, and a 1 h ramp down to 6°C (Fig. 1).

### Library preparation and sequencing

Pupae were collected at 29°C at 14:00 prior to treatment. The following day pupae were collected at 20°C at 12: 00 at the end of the warm pulse, and from both the FTR and CT treatments at 6°C at 15:00. Pupae were dissected from brood cells at incubation temperatures, immediately submerged in liquid nitrogen, and maintained at −80°C prior to messenger RNA (mRNA) extraction. mRNA was extracted using the Trizol protocol. Quality assessment and quantification were performed by Nanodrop and Qubit. RNA was shipped on dry ice to Georgia Genomics Facility for sequencing. Prior to sequencing quality was assessed using a Bioanalyzer. Paired-end libraries were generated from three replicates per time point. Illumina sequencing was performed on one high volume NextSeq 500 flowcell. Quality of the resulting sequence reads was assessed using FastQC (v0.11.7) (Andrews 2010). Overrepresented sequences and any remaining Illumina sequencing artifacts were removed using the BBDuk functions of the BBMap software suite (v38.18) (Bushnell 2014). Illumina data is archived at the NCBI Sequence Read Archive (BioProject: PRJNA528472).

### Differential expression analysis

**Fig. 2:**
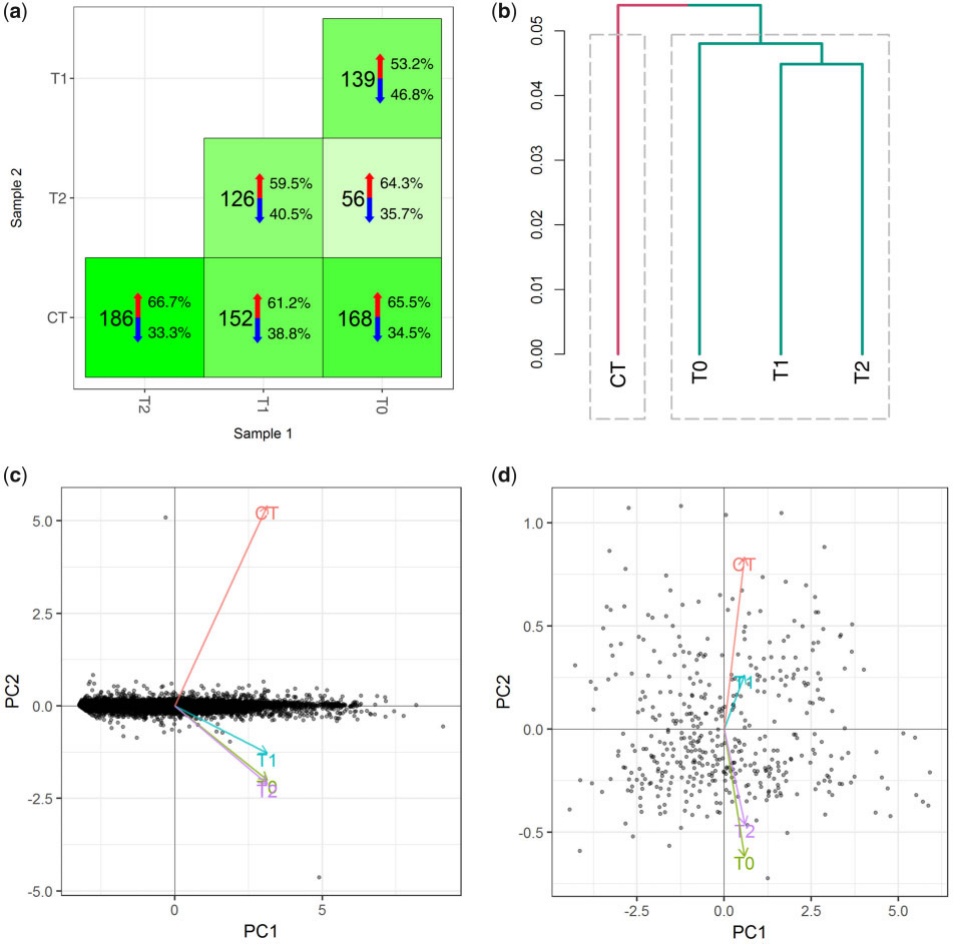
Summary of differential expression analysis of CT versus FTR treatments. Transcripts were identified as significant with a cutoff of α ≤ 0.05. The number of significant transcripts and the direction of expression were identified for all comparisons, with expression disproportionately up-regulated under FTR relative to CT (**a**), with the *x*-axis represented by the red arrow and the *y*-axis represented by the blue arrow. A dendrogram was constructed using the FPKM values of all annotated *M. rotundata* genes to determine the Jensen–Shannon distance between treatments and sample time points (**b**). Principle component analysis was performed on all genes (**c**) and the subset of significant genes (**d**).

Sequenced reads were aligned to the *M. rotundata* genome (accession: GCF_000220905.1) using Hisat2 (v2.1.0) (Kim et al. 2015). Mapped reads were quantified with Cufflinks (v2.2.1) (Trapnell et al. 2012) and assembled with the reference GTF annotation. Cuffdiff (v2.2.1) (Trapnell et al. 2012) was used to analyze gene expression with a threshold of (α ≤ 0.05) to determine significance. All subsequent analysis was performed in R (v3.4.2) (R Core Team 2017) and RStudio (v1.1.383) (RStudio Team 2016). The R package cummeRbund (v2.8.2) (Goff et al. 2013) was used to produce differential expression figures (Fig. 2a–d, Supplementary Fig. SF1). Jensen–Shannon distance between time points and treatments was assessed using the Fragments Per Kilobase of transcript per Million mapped reads (FPKM) values of the full gene set (Fig. 2b). Principle component analysis was performed using cummeRbund (v2.8.2) (Goff et al. 2013) on the FPKM values of both the full gene set and the differentially expressed transcripts (Fig. 2c, d).

Orthologous *M. rotundata* genes were identified in the *Apis mellifera* genome (version Amel_HAv3.1, accession: GCA_003254395.2) using standalone NCBI-BLAST+ (v2.8.1) (Camacho et al. 2009) and a Python (v2.7) reciprocal best hit script to parse tabular results by score. Enrichment analysis was performed on the resulting orthologs. Gene Ontology (GO) term enrichment, Kyoto Encyclopedia of Genes and Genomes (KEGG) pathway enrichment, and protein function enrichment were determined using the Database for Annotation, Visualization, and Integrated Discovery (DAVID v6.8) (Huang et al. 2007, 2009a, 2009b) using an EASE score of (α ≤ 0.05). Enrichment was analyzed by comparison and direction of expression. GO terms were identified using the functional annotation assignments of significant transcripts in DAVID. The functional annotation clustering tool in DAVID using InterPro annotation was used to cluster transcripts with similar features and functions. Clusters were combined using higher-level terms. Significance of combined clusters was established by Fisher’s method for combined probability using the R package metap (v1.1) (Brown 1975; Kost and McDermott 2002; Dewey 2019) and enrichment scores were calculated using a weighted mean. Additional tools used to identify protein function include NCBI, HymenopteraMine (Elsik et al. 2016), OrthoDB (v10) (Kriventseva et al. 2019), and FlyBase (Elsik et al. 2016; Agarwala et al. 2018; Kriventseva et al. 2019; Thurmond et al. 2019).

Differentially expressed transcripts under FTR in *M. rotundata* identified by Torson et al. (2015, 2017) were retrieved from the original publications. Annotation of the *M. rotundata* genome in the most recent genome release was applied by sequence alignment using NCBI-BLAST+ (v2.8.1) (Camacho et al. 2009) to facilitate comparison between studies. Significant transcripts found in multiple studies were identified using R (v3.4.2) (R Core Team 2017) and DB Browser for SQLite (v3.10.1).

## Results

### Read mapping and differential expression analysis

Sequencing generated 464.6 million, 126 base pair paired-end reads averaging 38.7 million reads per sample (Table 1). After quality trimming and removal of over-represented sequences, an average of 27.8 million reads (71.7%) per sample mapped to the *M. rotundata* genome (accession: GCA_000220905.1) leaving 11.0 million reads (28.3%) unmapped (Table 1). Analysis of gene expression identified 827 significant differentially expressed transcripts between all pairwise comparisons (Fig. 2a, Supplementary Table S1). 442 (53.45%) transcripts are unique and 385 (46.55%) are shared by two or more comparisons. Gene expression is disproportionately up-regulated under FTR compared to CT regardless of temperature (Fig. 2a): 65.5% of transcripts were up-regulated at T0, 61.2% during the warm pulse (T1), and 66.7% after returning to 6°C (T2) versus CT. The largest number of differentially expressed transcripts occurs at T2 versus CT. These samples were collected at the same time and temperature indicating persistent effects of the FTR warm pulse. Among FTR time points, expression is up-regulated at higher temperatures, 64.3% at T0 and 59.5% at T1, versus T2, although T0/T2 have the fewest differentially expressed transcripts (Fig. 2a). Jensen–Shannon distance of FPKM values shows progressive divergence from CT over time (Fig. 2b). Principle component analysis of all transcripts (Fig. 2c) and the subset of significant transcripts (Fig. 2d) show a distinct difference in expression profile between treatments and greater similarity between T0 at 29°C and T2 at 6°C than T1 at 20°C during the warm pulse (Fig. 2c).

**Table 1:** Sequencing and mapping statistics

Sample	Treatment	Reads	Mapped		Unmapped	
T0-1	FTR (29°C)	37,062,935	27,531,929	(74.3%)	9,531,006	(25.7%)
T0-2	FTR (29°C)	36,663,032	26,561,683	(72.4%)	10,101,349	(27.6%)
T0-3	FTR (29°C)	37,589,676	25,144,257	(66.9%)	12,445,419	(33.1%)
T1-1	FTR (20°C)	33,339,558	27,135,877	(81.4%)	11,981,208	(35.9%)
T1-2	FTR (20°C)	39,256,402	29,176,358	(74.3%)	11,768,736	(30.0%)
T1-3	FTR (20°C)	34,693,423	29,481,740	(85.0%)	12,791,732	(36.9%)
T2-1	FTR (6°C)	39,939,573	27,576,476	(69.0%)	12,363,097	(31.0%)
T2-2	FTR (6°C)	39,816,866	26,927, 100	(67.6%)	12,889,766	(32.4%)
T2-3	FTR (6°C)	43,865,807	32,342,409	(73.7%)	11,523,398	(26.3%)
CT-1	CT (6°C)	39,117,085	25,909,089	(66.2%)	7,430,469	(19.0%)
CT-2	CT (6°C)	40,945,094	28,629,912	(69.9%)	10,626,490	(26.0%)
CT-3	CT (6°C)	42,273,472	26,643,691	(63.0%)	8,049,732	(19.0%)
	Total	464,562,923	333,060,521	(71.7%)	131,502,402	(28.3%)
	Mean	38,134,436	27,778,692	72.8%	11,282,618	(29.6%)

*Notes:* 126 base-pair paired-end Illumina reads were sequenced and mapped to the *M. rotundata* genome.

### Membrane fluidity, lipid synthesis and modification, and lipid transport under FTR

**Fig. 3:**
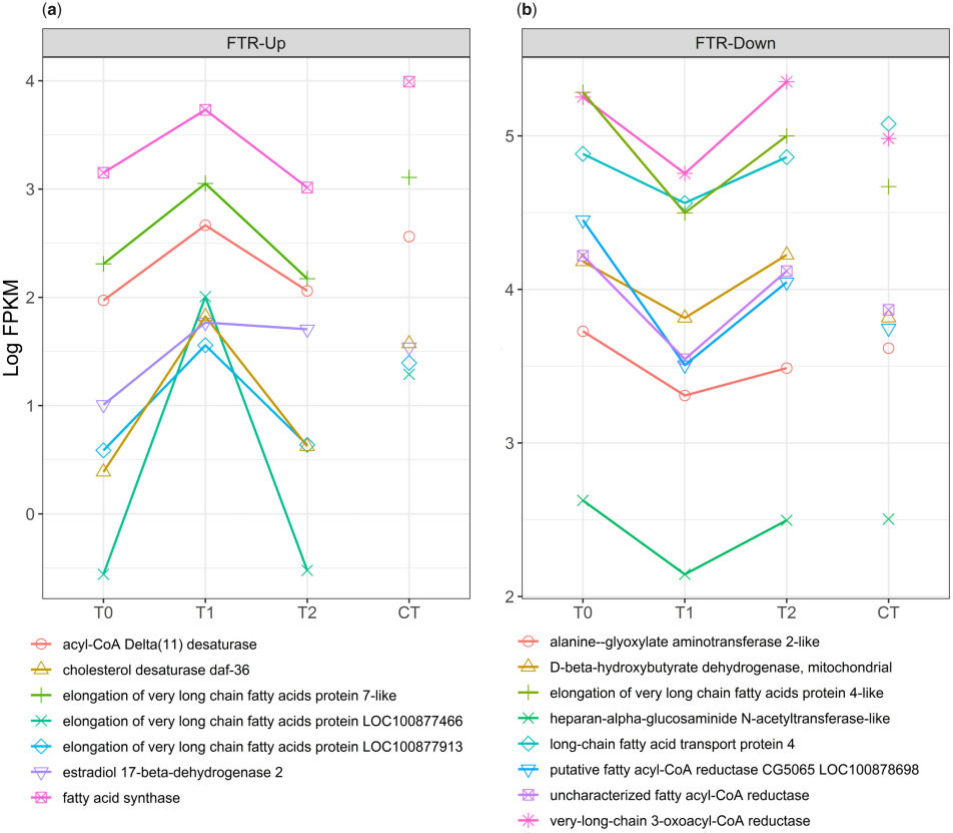
Expression of membrane component genes under FTR. Genes associated with membrane fluidity, lipid biosynthesis, and fatty acid modification are up-regulated (**a**) and down-regulated (**b**) during the warm pulse. During the warm pulse fatty acid synthase and fatty acid elongation enzymes are up-regulated and are down-regulated after the pulse with the exception of estradiol 17-beta-dehydrogenase 2 (a). Fatty acyl CoA, long-chain fatty acid transport protein 4, and a fatty acid elongation enzyme are down-regulated (b). T0-T2 represent expression over time under FTR versus CT.

**Fig. 4:**
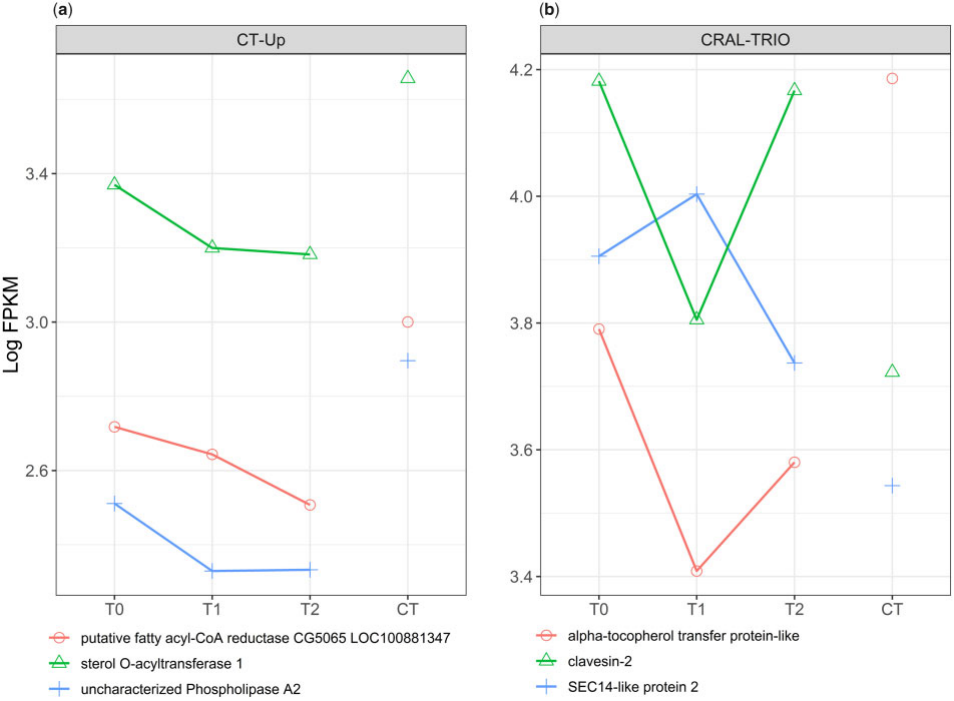
Expression of membrane components under CT and expression of CRAL-TRIO family enzymes. Some genes that affect membrane composition are significantly up-regulated under CT but are not affected by the warm pulse (**a**). CRAL-TRIO genes, intracellular membrane-bound transporters that affect fluidity by exchanging phospholipids, are differentially expressed at T1 or T2 versus CT (**b**). T0-T2 represent expression over time under FTR versus CT.

Genes associated with membrane fluidity (Figs 3, 4) are up-regulated (Fig. 3a) and down-regulated (Fig. 3b) during the warm pulse relative to T0. Genes directly involved in lipid biosynthesis and modification are among the most abundant. Fatty-acid synthase (LOC100878819) performs diverse lipid biosynthesis functions (Wakil 1989). GNS1/SUR4 family fatty acid elongation proteins (LOC100880416, LOC100877574, LOC100877913, LOC100877466) modify lipids and function to generate lipid diversity through interaction with fatty acyl-CoA enzymes (LOC100881347, LOC100878698, LOC100876260), serine kinases (LOC100877637, LOC100881145) aminotransferases (LOC100878030), and glycoside hydrolase-family enzymes (LOC100877705, LOC100879769, LOC100878475) (Holthuis and Menon 2014) (Fig. 3a, b). The cholesterol desaturase *neverland* (LOC100877176), Delta 11 acyl-CoA desaturase (LOC100881714), and desaturase/reductase enzymes (LOC100881714, LOC100881578, LOC100879632) modify fatty acid chains to influence membrane fluidity. Intracellular lipid transport proteins include two long-chain fatty acid transporters (LOC100883461, LOC100876568), CRAL-TRIO domain lipid-binding transporters (*SEC14-like, clavesin-2*, LOC413056, Fig. 4b) (Salama et al. 1990; Schaaf et al. 2008), and acytltransferases (LOC100878398, LOC100879483). Enzymes that affect membrane glycerophospholipid composition include alanine-glyoxylate aminotransferase 2-like (LOC100879339), which is up-regulated during the warm pulse and phospholipase A2 (LOC100877091), which is up-regulated under CT (Huang et al. 2007). ABC transporter G 20-like (LOC100877576), an adenosine triphosphate-binding transmembrane transporter associated with intracellular cholesterol and lipid transport, is down-regulated under CT.

### Oxidative stress

**Fig. 5:**
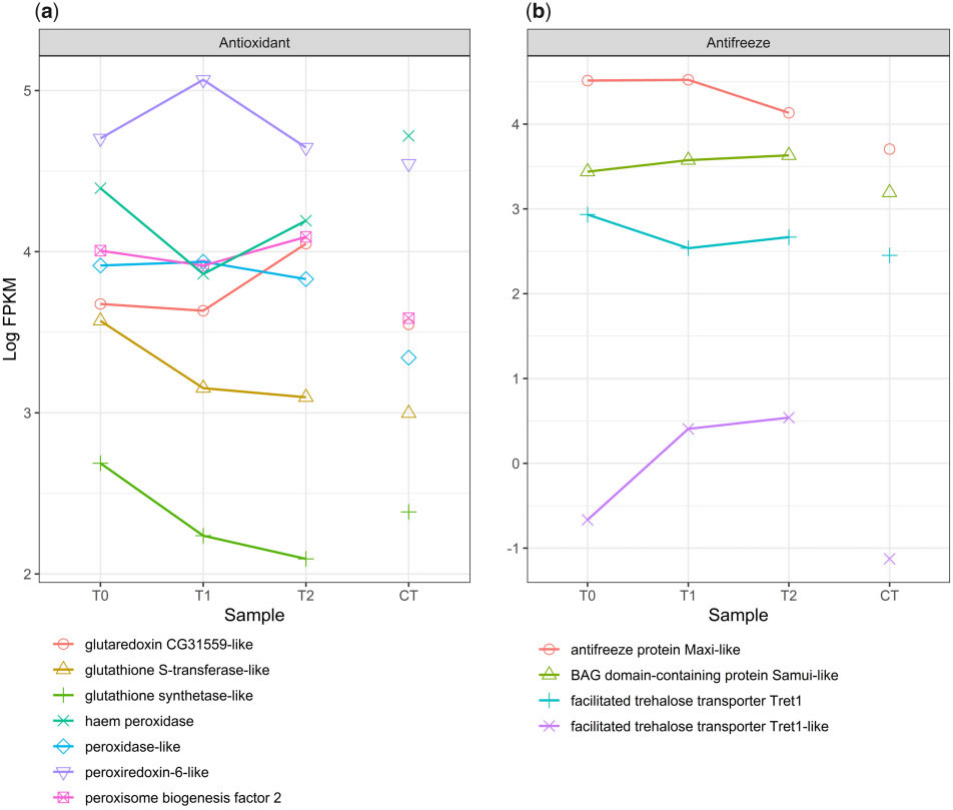
Antioxidant and antifreeze response to FTR. Glutathione synthase is down-regulated under FTR. Glutathione transferase expression declines under FTR and is not significantly different between T2 and CT. Peroxiredoxin-6 is up-regulated during the warm pulse and glutaredoxin expression increases after the pulse. Peroxidases and peroxisome biogenesis factor 2 are differentially expressed between FTR and CT treatments (**a**). Antifreeze protein Maxi-like is differentially expressed at T0 and T1 versus CT. Trehalose transporters are regulated in opposing directions. Expression of the temperature-associated chaperone *samui* is significantly different at T2 versus CT (**b**). T0-T2 represent expression over time under FTR versus CT.

The oxidative stress response (Fig. 5a) includes glutathione synthetase (LOC100876989) and glutathione S-transferase (LOC100876760), which generate the glutathione pool that buffers oxidative stress, and are significantly up-regulated at T0 versus CT. Glutathione synthetase expression decreases under FTR and is significantly up-regulated under CT versus T2. Peroxidases (LOC100875470, LOC105664053, LOC100882514) and enzymes that catalyze redox-reactions (LOC100875155, LOC100883439) were up-regulated under FTR (Fig. 5a). Three cytochrome P450 genes (LOC100883162, LOC100879963, LOC100880078) were up-regulated during the warm pulse. However, some genes that regulate the oxidation–reduction response were not differentially expressed, including superoxide dismutase.

### Reponse of cryoprotectant, ion transport, chitinase, and cuticle protein transcripts

**Fig. 6:**
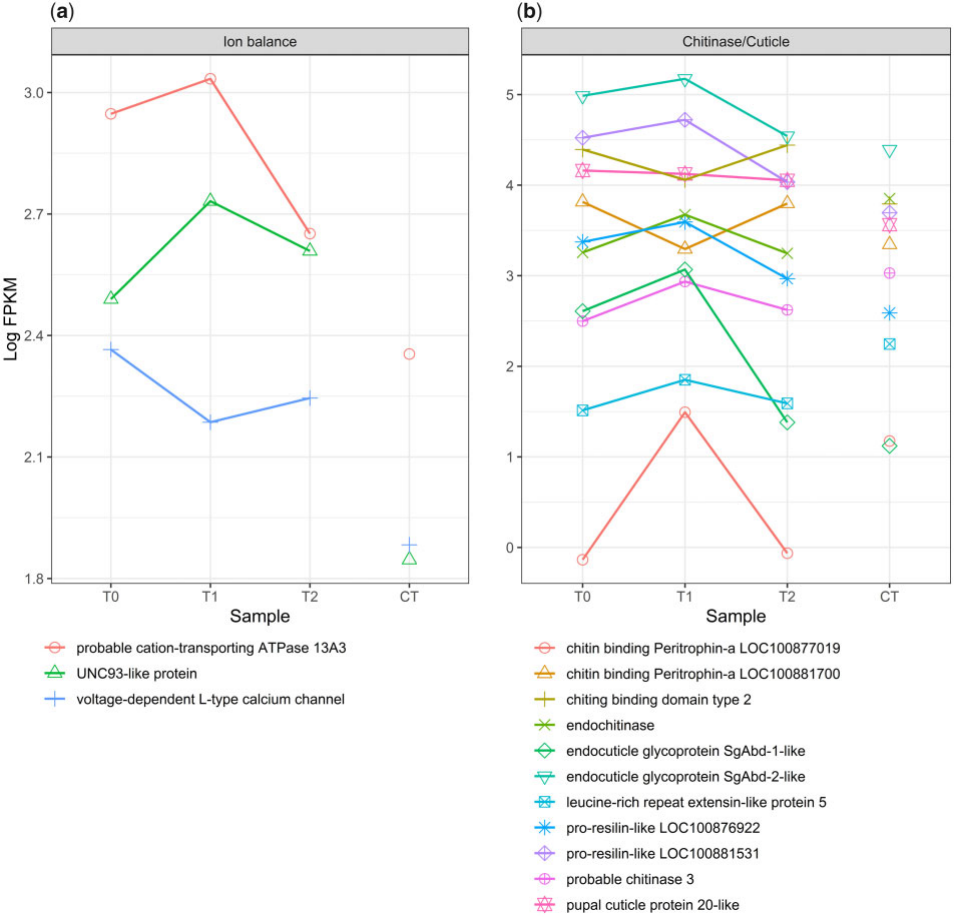
Ion channel, chitinase, and cuticle protein expression during FTR. Ion channel proteins respond to the warm pulse and are significantly down-regulated versus CT (**a**). Chitinase, chitin-binding proteins, and cuticle proteins are differentially expressed between FTR and CT treatments although the mechanism and function of this response is unknown (**b**). T0-T2 represent expression over time under FTR versus CT.

Genes with cryoprotectant functions were differentially expressed between treatments (Fig. 5b). Trehalose transport proteins (LOC100882177, LOC100878705) are expressed in opposing directions. Expression of antifreeze protein Maxi-like (LOC100879693) is maintained during the warm pulse but reduced under CT. The gene *samui* (LOC100881147) is up-regulated between treatments at T2 versus CT. Other known cryoprotectants were not differentially expressed, including genes that synthesize trehalose, glycerol or other polyols, and sorbitol. Three membrane-bound ion transport channel proteins (Fig. 6a), voltage-dependent L-type calcium channel (LOC100875269), probable cation-transporting ATPase 13A3 (LOC100876262) and potassium ion channel UNC93-like protein (LOC100883536), were down-regulated under CT. Chitinase enzymes (LOC100879953, LOC100878742) are down-regulated under FTR (Fig. 6b). Of four proteins with chitin-binding domains, two are down-regulated under FTR (LOC100879494, LOC100877019), two up-regulated (LOC100881700, LOC105662353). Cuticle proteins were all up-regulated under FTR (LOC100883766, LOC100880223, LOC100876922, LOC100881531, LOC100883648).

### Enrichment analysis

Analysis of significant transcripts using DAVID identified enriched clusters of genes with enriched GO terms, KEGG pathways, and protein features/functions. All differentially expressed transcripts in this study are summarized by protein function in Table 2. Categories with the largest number of transcripts include transcription factors and HOX genes, membrane-bound proteins, and protein kinases. Pathway enrichment analysis (Table 3) shows metabolic pathways and phenylalanine metabolism are elevated at T0 versus T1 and T2, indicating a reduction in metabolic activity after pupal development is interrupted that is maintained 24-h later. Fatty acid metabolism is down-regulated at T1. No pathways are enriched between T1 and T2, during and after the warm pulse, respectively. Between FTR and CT treatments, phenylalanine and tyrosine metabolism are up-regulated at T0. Two transcripts map to multiple down-regulated pathways associated with glycan metabolism and glycosphingolipid biosynthesis (Table 3). A comparison of warm (T0, T1) and cold (T2, CT) shows enrichment of the Hippo signaling pathway (fold enrichment [FE] = 3.9, *P* = 0.017) including the HOX gene *homothorax*, *dachsous* which mediates imaginal disc development and cellular adhesion, and *expanded* which regulates Hippo signaling and cell proliferation during development (Willecke et al. 2008; Halder and Johnson 2011).

**Table 2:** Protein function annotation clustering of significant transcripts

Function summary	*n*	χ^2^	*P*	ES
Lipid metabolism	8	84.97	<0.0001*	2.05
Chitin binding	9	43.18	<0.0001*	1.88
Transcription, DNA-binding, HOX genes	43	234.9	<0.0001*	1.12
Pyridoxal phosphate binding	5	25.24	0.005*	1.1
CRAL-TRIO binding domain	3	14.62	0.023*	1.06
Membrane components	49	17.06	0.029*	0.17
Serine protease	3	23.73	0.095	0.64
Glycoside hydrolase	3	8.86	0.35	0.48
Protein kinase activity	26	26.47	0.33	0.48
Major facilitator superfamily	7	6.47	0.37	0.47
Leucine-rich repeat	5	5.28	0.51	0.38

*Notes:* All differentially expressed transcripts were clustered by InterPro protein features and functions. Clusters with similar functions were combined to summarize overall transcript function with *n* representing the number of unique transcript identities in each group. χ^*2*^ and *P-values* use Fisher’s method for testing combined probability to determine significance. ES indicates combined enrichment scores by weighted mean.

**Table 3:** Pathway enrichment of significant differentially expressed transcripts

Comparison	Term	Description		FE	*n*	*P*
T0 (29°C) vs. CT (6°C)	ame00360	Phenylalanine metabolism	↑	70.43	2	0.023
	ame00350	Tyrosine metabolism	↑	46.96	2	0.035
T1 (20°C) vs. CT (6°C)	ame00604	Glycosphingolipid biosynthesis	↓	105.65	2	0.016
	ame00531	Glycosaminoglycan degradation	↓	40.63	2	0.042
	ame00511	Other glycan degradation	↓	37.73	2	0.046
T2 (6°C) vs. CT (6°C)	None					
T0 (29°C) vs. T1 (20°C)	ame01100	Metabolic pathways	↑	2.18	7	0.034
	ame00360	Phenylalanine metabolism	↑	42.26	2	0.042
	ame01212	Fatty acid metabolism	↓	28.55	2	0.05
T1 (20°C) vs. T2 (6°C)	None					
T0 (29°C) vs. T2 (6°C)	ame01100	Metabolic pathways	↑	3.12	5	0.01
	ame00360	Phenylalanine metabolism	↑	84.52	2	0.019
	ame00380	Tryptophan metabolism	↑	36.75	2	0.043

*Notes:* KEGG pathway enrichment using *A. mellifera* orthologs. Enrichment was determined by mapping up-regulated or down-regulated transcripts to KEGG pathways for each comparison with a cutoff of α ≤ 0.05. The direction of expression is indicated by the arrow, with FE, and the number (*n*) of unique transcripts mapping to each pathway.

GO term enrichment shows development resumes during the warm pulse (Table 4). Multiple developmental transcription factors including *drop*, *distal-less*, *engrailed*, *homothorax*, and the co-repressors *slp1*, *groucho*, and *hairy* are up-regulated under FTR. This pattern of expression is maintained across all FTR time points including T0 where these transcripts are significantly up-regulated, but the associated GO term is not significantly enriched (FE = 3.38, *P* = 0.071). Analysis of enriched GO terms under FTR shows decreased expression of membrane-bound transport proteins during the warm pulse (Table 5). Fatty acyl-CoA reductase activity increased at T1, during the warm pulse, and decreased at T2. Fatty acid biosynthesis activity decreased at T1 and increased at T2. Frequently occurring GO terms are found in Supplementary Table S2. Enriched InterPro protein feature/function terms are summarized in Tables 6 and 7. Transcripts categorized as hemolymph juvenile hormone binding (Table 6), *takeout-like* and *circadian clock-controlled protein*, belong to the *takeout* superfamily associated with circadian rhythm and feeding behavior.

**Table 4:** GO term enrichment of significant differentially expressed transcripts under FTR versus CT

Sample		Term	Description		FE	*n*	*P*
T0 (29°C)							
	BP	GO: 0016021	Aromatic amino acid metabolic process	↑	82.18	2	0.022
	MF	GO: 0042302	Structural constituent of cuticle	↑	16.56	5	<0.001
	MF	GO: 0005506	Iron ion binding	↑	8.03	3	0.05
	BP	GO: 0006633	Fatty acid biosynthetic process	↓	31.8	3	<0.01
	MF	GO: 0102337	3-oxo-cerotoyl-CoA synthase activity	↓	80.88	3	<0.001
T1 (20°C)							
	BP	GO: 0006355	Regulation of transcription	↑	7.74	7	<0.0001
	CC	GO: 0005634	Nucleus	↑	2.73	7	0.028
	MF	GO: 0042302	Structural constituent of cuticle	↑	16.56	5	<0.001
	MF	GO: 0043565	Sequence-specific DNA binding	↑	4.56	5	0.02
	MF	GO: 0030170	Pyridoxal phosphate binding	↓	21.85	3	<0.01
T2 (6°C)							
	BP	GO: 0006355	Regulation of transcription	↑	5.05	6	0.004
	MF	GO: 0043565	Sequence-specific DNA binding	↑	5.01	6	<0.01
	BP	GO: 0006633	Fatty acid biosynthetic process	↓	28.62	3	<0.01
	BP	GO: 0006355	Regulation of transcription	↓	59.31	3	<0.001

*Notes:* GO term enrichment of FTR versus CT treatments with a cutoff of α ≤ 0.05. Direction of expression under FTR is indicated by the arrow. The number of transcripts (*n*) and FE are shown for each term.

**Table 5:** GO term enrichment of significant differentially expressed transcripts under FTR

Comparison		Term	Description		FE	*n*	*P*
T0 (29°C) vs. T1 (20°C)							
	CC	GO: 0016021	Integral component of membrane	↑	1.63	12	0.017
	MF	GO: 0030170	Pyridoxal phosphate binding	↑	21.85	3	<0.01
	MF	GO: 0080019	fatty-acyl-CoA reductase activity	↑	69.2	2	0.026
	BP	GO: 0006633	Fatty acid biosynthetic process	↓	19.08	3	<0.01
	BP	GO: 0006355	Regulation of transcription, DNA-templated	↓	4.72	4	0.04
T1 (20°C) vs. T2 (6°C)							
	BP	GO: 0006633	Fatty acid biosynthetic process	↑	40.88		0.002
	MF	GO: 0102337	3-oxo-cerotoyl-CoA synthase activity	↑	74.14	3	<0.001
	MF	GO: 0102336	3-oxo-arachidoyl-CoA synthase activity	↑	74.14	3	<0.001
	MF	GO: 0102338	3-oxo-lignoceronyl-CoA synthase activity	↑	74.14	3	<0.001
	MF	GO: 0080019	fatty-acyl-CoA reductase activity	↓	148.28	2	0.012
T0 (29°C) vs. T2 (6°C)							
	MF	GO: 0030170	Pyridoxal phosphate binding	↑	31.22	2	0.05

*Notes:* Enrichment of GO terms across FTR time points with a cutoff of α ≤ 0.05. Direction of expression in each comparison is indicated by the arrow. The number of transcripts (*n*) and FE are shown for each term.

**Table 6:** Protein function enrichment of significant transcripts under FTR versus CT

Sample	Term	Description		FE	*n*	*P*
T0 (29°C)						
	IPR000618	Insect cuticle protein	↑	16.47	5	<0.001
	IPR001273	Aromatic amino acid hydroxylase	↑	125.14	2	0.017
	IPR019773	Tyrosine 3-monooxygenase-like	↑	125.14	2	0.017
	IPR018301	Aromatic amino acid hydroxylase, iron/copper	↑	125.14	2	0.017
	IPR002076	GNS1/SUR4 membrane protein	↓	86.4	3	<0.001
T1 (20°C)						
	IPR000618	Insect cuticle protein	↑	17.69	5	<0.001
	IPR013087	Zinc finger C2H2-type/integrase DNA-binding	↑	4.73	5	0.02
	IPR001680	WD40 repeat	↑	4.48	5	0.023
	IPR001507	Zona pellucida domain	↓	52.79	2	0.036
T2 (6°C)						
	IPR013087	Zinc finger C2H2-type/integrase DNA-binding	↑	3.93	5	0.036
	IPR002076	GNS1/SUR4 membrane protein	↓	62.21	3	<0.001
	IPR010562	Hemolymph juvenile hormone binding	↓	41.47	2	0.045

*Notes:* Enrichment of InterPro protein functions/features between FTR and CT treatments with a cutoff of α ≤ 0.05. Direction of expression under FTR is indicated by the arrow. The number of transcripts (*n*) and FE are shown for each term.

**Table 7.** Protein function enrichment of significant transcripts under FTR

Comparison	Term	Description		FE	*n*	*P*
T0 (29°C) vs. T1 (20°C)						
	IPR015422	Pyridoxal phosphate-dependent transferase	↑	20.74	3	<0.01
	IPR013120	Male sterility, NAD-binding	↑	60.48	2	0.03
	IPR026055	Fatty acyl-CoA reductase	↑	60.48	2	0.03
	IPR002076	GNS1/SUR4 membrane protein	↓	51.84	3	0.001
	IPR001356	Homeodomain	↓	12.1	4	<0.01
	IPR017970	Homeobox, conserved site	↓	10.67	3	0.03
T1 (20°C) vs. T2 (6°C)						
	IPR002076	GNS1/SUR4 membrane protein	↑	77.76	3	<0.001
	IPR010562	Hemolymph juvenile hormone binding	↑	77.76	3	<0.001
	IPR001611	Leucine-rich repeat	↑	12.23	3	0.022
	IPR016040	NAD(P)-binding domain	↓	19.67	5	<0.0001
	IPR002347	Glucose/ribitol dehydrogenase	↓	37.22	3	0.002
	IPR013120	Male sterility, NAD-binding	↓	120.97	2	0.015
	IPR026055	Fatty acyl-CoA reductase	↓	120.97	2	0.015
T0 (29°C) vs. T2 (6°C)						
	IPR020846	Major facilitator superfamily domain	↓	19.2	3	0.008
	IPR013761	Sterile alpha motif/pointed domain	↓	57.6	2	0.03
	IPR005828	General substrate transporter	↓	37.51	2	0.046

*Notes:* Enrichment of InterPro protein functions/features across FTR time points with a cutoff of α ≤ 0.05. Direction of expression in each comparison is indicated by the arrow. The number of transcripts (*n*) and FE are shown for each term.

### Identification of conserved transcripts from previous FTR treatments

Transcripts expressed after one FTR cycle that were identified in Torson et al. (2015, 2017) are summarized in Table 8. Transcript sequences found in short-term interrupted pupal development (Torson et al. 2017) and long-term survival where FTR-treated bees experience significantly lower mortality over months of incubation (Torson et al. 2015) were retrieved from the original publications. The *M. rotundata* genome had been annotated in the intervening time and transcripts from these studies received updated annotation by sequence alignment which removed redundant sequences. The sequence content of the *M. rotundata* genomes used in this and previous studies did not differ and alignments matched 100% of nucleotide identities.

**Table 8:** Selected differentially expressed transcripts across FTR studies

Gene set	RefSeq-RNA	Short name	Gene name
**2017—Up in FTR**			
	XM_003708250	LOC100881147	BAG domain-containing protein Samui-like
	XM_003708002	LOC100877637	Serine/threonine-protein kinase SIK3-like
	XM_003699760	LOC100880515	Bone morphogenetic protein receptor type-1B
	XM_012282783	LOC100881489	Nuclear hormone receptor FTZ-F1
	XM_012281076	LOC105661976	CCAAT/enhancer-binding protein-like
**2017—Down in FTR**			
	XM_012288926	LOC100880270	Transmembrane protease serine 9-like
	XM_003700471	LOC100877574	Elongation of very long chain fatty acids protein 7-like
	XM_003703558	LOC100878819	Fatty acid synthase
	XM_003700398	LOC100878398	Heparan-alpha-glucosaminide N-acetyltransferase-like
	XM_012292966	LOC100880205	Phosphodiesterase epsilon-1-like
	XM_003702488	LOC100880638	Aquaporin AQPcic-like
	XM_003703107	LOC100875155	Peroxiredoxin-6-like
	XM_003704386	LOC100879301	Alpha-amylase-like
	XM_003702306	LOC100879369	Carboxypeptidase M-like
	XM_012287600	LOC100882780	Serine proteinase stubble
	XM_003701259	LOC100882217	Protein yellow-like
	XM_003704962	LOC100880044	Transmembrane domain-containing protein 2-like
	XM_012286750	LOC100881714	Acyl-CoA Delta
	XM_003702234	LOC100880821	Glutamic acid-rich protein
	XM_003707059	LOC100879468	Phenoloxidase 2
	XM_012286098	LOC105662570	Histidine-rich glycoprotein-like
	XM_012284520	LOC100877204	Vitellogenin-like
	XM_012284233	LOC100878705	Facilitated trehalose transporter Tret1-like
**2015—NovSTR NovFTR**			
	XM_003700977	LOC100878060	Hemolymph lipopolysaccharide-binding protein-like
	XM_003700471	LOC100877574	Elongation of very long chain fatty acids protein 7-like
	XM_003704386	LOC100879301	Alpha-amylase-like
	XM_003700707	LOC100875958	Cytochrome P450 4g15-like
**2015—SeptFTR NovFTR**			
	XM_003702880	LOC100878030	Aminomethyltransferase, mitochondrial
	XM_003706384	LOC100877030	Aminotransferase, mitochondrial-like
	XM_003700707	LOC100875958	Cytochrome P450 4g15-like
	XM_003703558	LOC100878819	Fatty acid synthase
**2015—SeptSTR NovSTR**			
	None		
**2015—SeptSTR SeptFTR**			
	XM_003702196	LOC100876503	Mitochondrial amidoxime-reducing component 1
	XM_003707130	LOC100877705	Beta-galactosidase-like
	XM_012286145	LOC100882514	Peroxidase-like
	XM_003702880	LOC100878030	Aminomethyltransferase, mitochondrial
	XM_003700707	LOC100875958	Cytochrome P450 4g15-like
	XM_012283276	LOC100879483	Sterol *O*-acyltransferase 1

*Notes:* Differentially expressed transcripts were retrieved from Torson et al. (2015, 2017) and annotated by sequence alignment to the current genome release (Torson et al. 2015, 2017). Significant transcripts shared between studies are summarized here.

Torson et al. (2017) identified transcripts differentially expressed during interrupted pupal development after seven FTR cycles (Torson et al. 2017). Of the 256 differentially expressed transcripts identified, 86 (23.76%) were found to be significant in this study. The direction of expression of shared transcripts were disproportionately down-regulated (*n* = 72, 83.72%) versus up-regulated (*n* = 14, 16.82%). This is a result of the disproportionate 71.74% down-regulation present in the original study. Common transcripts regulate transcription during development, are involved in metabolic processes, or are membrane-bound transport or signaling proteins (Table 8).

Torson et al. (2015) identified 215 transcripts under long-term FTR when mortality begins to diverge and a protective effect of FTR versus CT is observed. Of these 29 of 256 (11.33%) were shared between studies. *Megachile rotundata* were sequenced as pre-pupa, an earlier stage of development, which corresponds to the lack of developmental transcription factors and metabolic genes from the list of shared transcripts (Table 8). The remaining identities include orthologs for cytochrome p450, peroxidase, and mitochondrial amidoxime-reducing component which function as chaperones, the oxidative stress response, and in DNA-repair.

## Discussion

Ectotherms experience daily temperature variation as well as broad seasonal variation in the range and magnitude of temperature change. With a limited ability to regulate internal temperature, insects have multiple adaptations that allow them to survive fluctuations in temperature. Temperature variation and FTR improve survival and longevity in many species (Rinehart et al. 2011, 2013, 2016; Colinet et al. 2015, 2018). Previous studies indicated that brief exposures to fluctuating temperatures were sufficient to establish differential gene expression patterns (Torson et al. 2017) that may provide a protective effect that reduces mortality observed over longer periods of exposure (Torson et al. 2015). Our objective was to determine the transcriptional response to a single FTR pulse. We compare the response over one 24-h FTR cycle to storage under CT. We established that one FTR cycle is sufficient to cause differential expression of transcripts associated with the repair of cell membrane damage, restoration of ion homeostasis, and response to oxidative stress. We identify individual genes as well as enrichment of pathways, GO terms, and protein functions before, during, and after the warm pulse. We compared these results with previous studies on the same organism and two life stages and identified a shared response.

### Membrane composition responds rapidly to temperature

Insects possess highly diverse lipid species and enzymes that modify lipids in cell and intracellular membranes that facilitate rapid response to temperature fluctuations (Hazel 1995; Los and Murata 2004; Kimura et al. 2016). Membrane components were the largest cluster of genes by general function, significantly enriched pathways, GO terms, and protein functions/features. Lipid biosynthesis and fatty acid metabolism respond to FTR but individual genes are regulated in opposing directions (Fig. 3a, b, Table 3). 3-oxo-cerotoyl-CoA synthase activity, a product of fatty acyl-CoA synthase, is down-regulated under FTR versus CT (Table 4). Among FTR time points, two sets of membrane components and lipid synthesis/modification genes are regulated in opposite directions (Tables 5, 7). Although some have functions unrelated to membrane composition, many groups identified in Table 2 directly or indirectly influence membranes through lipid species diversity, modification of fatty acids, desaturase/reductase activity, lipid and cholesterol transport, and potential mobilization of cryoprotectants to support membrane integrity. Fatty-acid synthase is a highly versatile enzyme that functions in multiple lipid biosynthesis pathways (Wakil 1989). GNS1/SUR4 family fatty acid elongation proteins generate diverse lipid species by creating precursors of ceramide in combination with serine metabolism, glycosphingolipids, and sphingolipids (Holthuis and Menon 2014). Phospholipids are synthesized through elongation and desaturation of fatty-acid synthase intermediate products, and addition of acyl CoA, cholesterol, and glycerol (Holthuis and Menon 2014). Multiple components of these pathways were found to be differentially expressed during exposure to FTR, indicating that FTR affects membrane composition and may restore membrane function through the synthesis and modification of membrane components.

In addition to lipid biosynthesis we found enzymes that influence membrane fluidity through desaturase/reductase activity. These enzymes are known to have intracellular membrane functions and are localized in endoplasmic reticulum, Golgi apparatus, or mitochondria, although the function of these enzymes has been shown to have diversified in some arthropods (Salama et al. 1990; Schaaf et al. 2008; Smith and Briscoe 2015; Kriventseva et al. 2019). Our results support the hypothesis that FTR exposure provides a recovery period that restores intracellular and cell membrane fluidity by modifying membrane composition, synthesis of diverse lipid species, and production of lipid transport proteins (Colinet et al. 2018). Additionally, loss of ion gradients through membrane phase transitions and oxidative damage to membranes have been hypothesized to accumulate under CT and may be a primary mechanism of increased survival under FTR (Colinet et al. 2018). However, our ability to make broad conclusions is limited by the fact that this study focuses on the whole organism response, and cannot differentiate between physiological processes at the organ level. Our results demonstrate that a 1-h exposure to a warm temperature is sufficient to activate the repair and modification of membranes. These mechanisms had been identified by other FTR studies in insects (Torson et al. 2015, 2017; Colinet et al. 2016), but were not known to operate under brief temperature exposures.

### Oxidative stress response, cryoprotectants, and chitinases

Under FTR, genes that respond to oxidative stress are up-regulated (Fig. 5a), including the cytochrome P450-family, peroxidases/peroxisomes, and redoxin enzymes. Expression of glutathione synthase and glutathione S-transferase S4, which maintain the glutathione pool for the removal of reactive oxygen species was significant (Felton and Summers 1995; Schafer and Buettner 2001). Superoxide dismutase was not differentially expressed between treatments or time points. The incubation at 6°C between collection of T0 and CT is 24 h and reactive oxygen species may not accumulate enough to induce a transcriptional response. Expression of some antioxidants like superoxide dismutase is a tissue-level response (Terada 2006; Wang et al. 2018) that may not be detectable at the whole-organism level.

Insects respond to cold stress by production of antifreeze proteins, cryoprotectant sugars, and polyols, which lower freezing temperatures and stabilize membranes (Sinclair et al. 2003). Trehalose synthase and enzymes that synthesize glycerol or sorbitol cryoprotectants are not differentially expressed. However, trehalose transport proteins are expressed at higher levels under FTR (Fig. 5b) which may indicate that trehalose is being mobilized in response to cold. Antifreeze protein Maxi-like is up-regulated under FTR versus CT and expression is maintained during the warm pulse, but is lower at T2 so it cannot be determined if expression is influenced by FTR.

Chitinase enzymes, chitin-binding proteins, and cuticle proteins have been identified as differentially expressed in multiple FTR and cold stress studies in several species (Colinet et al. 2007; Clark et al. 2009; Yocum et al. 2009; Torson et al. 2015, 2017). Expression of these transcripts appears to be a common feature in transcriptomic studies of FTR but they have not been specifically investigated. Because chitin is expressed in the midgut and the midgut reacts to many forms of stress, the mechanism is often hypothesized as midgut atrophy or repair (Yocum et al. 2009; MacRae 2010).

### Development resumes during the warm pulse

Genes that regulate development are significantly up-regulated after the warm pulse versus CT. Transcription factors in multiple families including HOX genes, Zn-fingers, WD40 repeats, and winged-helix that regulate gene expression are present. Growth factors, genes that coordinate cell proliferation and differentiation, cell signaling, and hormone-binding are expressed under FTR. We see a rapid transcriptomic response to the warm pulse with the production of developmental transcription factors. The large number of transcription factors up-regulated during the warm pulse after 20 h of interrupted development, indicate that these transcripts are in circulation for a relatively brief time. The 1-h pulse appears to be sufficient time above the developmental temperature threshold to resume production of these transcripts, although it is unknown if the warm pulse is long enough for development to progress beyond maintenance of the transcriptional response.

### Shared mechanisms of FTR

Genes and pathways in transcriptional response to FTR in *M. rotundata* were identified in previous studies, indicating the mechanisms that improve survival and reduce sub-lethal effects under FTR emerge quickly and are maintained over long periods of time in different life stages (Torson et al. 2015, 2017). The transcript *samui* was upregulated under FTR in both our study and Torson et al. (2017). Samui has been proposed as the trigger to end diapause in *Bombyx mori* (Moribe et al. 2001, 2002) and has been associated with diapause termination in *M. rotundata* (Yocum et al. 2018). The bees in this study had already terminated diapause prior to the start of the experiment, so the function of *samui* in this study is not directly related to diapause. However, *samui* may function as a molecular chaperone because it is in the BAG protein family (Doong et al. 2002; Kabbage and Dickman 2008) and may broadly function to maintain cellular processes during cold exposure.

## Conclusions

Our results establish that *M. rotundata* responds rapidly to temperature changes at the transcript level, and that these responses last beyond the initial exposure. The transcriptomic responses to FTR include genes associated with cell and intracellular organelle membranes that affect membrane composition and fluidity, ion homeostasis, oxidative stress, and antifreeze proteins. After a single 1-h exposure at 20°C, we found transcripts identified in previous studies associated with recovery from indirect chill injury. The brief exposure to warmth provided by FTR is enough to resume production of developmental transcription factors, as evidenced by transcripts associated with morphogenesis, and this effect persists even after a return to cold exposure. Finally, we identify the gene *Samui*, which seems to be a gene associated with a tolerance of cold exposure across studies.

## Authors’ contributions

J.P.R., G.D.Y., A.S.T., and D.M. designed the research plan. J.P.R. and G.D.Y. obtained the animals. Tissue collection was performed by A.S.T. and G.D.Y. at the USDA ARS Edward T. Schafer Agricultural Research Center. Gene expression analysis was performed by D.M. and T.J.A. Enrichment analysis and additional statistical analysis were performed by D.M. All authors helped write and edit the manuscript. All authors contributed to and approve the content of the final manuscript.

## Funding

This work was supported by funding from the United States Department of Agriculture, Agricultural Research Service, National Science Foundation Established Program to Stimulate Competitive Research (NSF-EPSCoR-1826834), the North Dakota State University College of Science and Mathematics and the Department of Biological Sciences.

## Availability of data and materials

The dataset supporting the conclusions of this article is included within the article (and its additional files). Sequence reads associated with the mRNA sequencing analysis are archived at NCBI (BioProject: PRJNA528472).

## Supplementary data

Supplementary data available at *ICB* online.

## Supplementary Material

icz096_Supplementary_Data

## Notes

From the symposium “Stress phenotype: linking molecular, cellular, and physiological stress responses to fitness” presented at the annual meeting of the Society for Integrative and Comparative Biology, January 3–7, 2019 at Tampa, Florida.
