# Supplementary material for: Immediate Transcriptional Response to a Temperature Pulse under a Fluctuating Thermal Regime
Source: Integr Comp Biol. 2019 Jun 7;59(2):320–37. doi: 10.1093/icb/icz096 (PMC6703998; doi:10.1093/icb/icz096)
Supplement: icz096_Supplementary_Data [file icz096_supplementary_data.zip › icb-2019-0064-File020.pdf]

009 - Single Pulse FTR - G - annotation only

Dacotah

December 14, 2018

```
cuff <- readCufflinks("../cuffdiff-big-G/")
runInfo(cuff)
```

```
##           param
## 1      cmd_line
## 2       version
## 3  SVN_revision
## 4 boost_version
##
value
## 1 cuffdiff -p 32 -v -o cuffdiff-G ./cuffmerged-G/merged.gtf -L T0,T1W,T2C,T0STR
../T0-A_sorted.bam,../T0-B_sorted.bam,../T0-C_sorted.bam ../T1WARM-A_sorted.bam,../
T1WARM-B_sorted.bam,../T1WARM-C_sorted.bam ../T1COLD-A_sorted.bam,../T1COLD-B_sorte
d.bam,../T1COLD-C_sorted.bam ../T1STR-A_sorted.bam,../T1STR-B_sorted.bam,../T1STR-C
_sorted.bam
## 2
2.2.1
## 3
4237
## 4
104700
```

```
replicates(cuff)
```

```
##                       file sample_name replicate rep_name total_mass
## 1      ../T0-A_sorted.bam          T0         0     T0_0    8512250
## 2      ../T0-B_sorted.bam          T0         1     T0_1    7822460
## 3      ../T0-C_sorted.bam          T0         2     T0_2    8315740
## 4  ../T1WARM-A_sorted.bam         T1W         0    T1W_0    8894250
## 5  ../T1WARM-B_sorted.bam         T1W         1    T1W_1    8852520
## 6  ../T1WARM-C_sorted.bam         T1W         2    T1W_2    8133370
## 7  ../T1COLD-A_sorted.bam         T2C         0    T2C_0    9033360
## 8  ../T1COLD-B_sorted.bam         T2C         1    T2C_1    8095240
## 9  ../T1COLD-C_sorted.bam         T2C         2    T2C_2   10416900
## 10  ../T1STR-A_sorted.bam       T0STR         0  T0STR_0    7760470
## 11  ../T1STR-B_sorted.bam       T0STR         1  T0STR_1    9645980
## 12  ../T1STR-C_sorted.bam       T0STR         2  T0STR_2    8513490
##    norm_mass internal_scale external_scale
## 1    8613340       0.989264              1
## 2    8613340       0.873376              1
## 3    8613340       0.943510              1
## 4    8613340       0.975793              1
## 5    8613340       1.129550              1
## 6    8613340       0.946387              1
## 7    8613340       1.104510              1
## 8    8613340       0.975137              1
## 9    8613340       1.070570              1
## 10   8613340       0.894246              1
## 11   8613340       1.197990              1
## 12   8613340       1.000200              1
```

```
samples(genes(cuff))
```

```
## [1] "T0"    "T1W"   "T2C"   "T0STR"
```

```
disp <- dispersionPlot(genes(cuff))
disp
```

```
## Warning: Transformation introduced infinite values in continuous x-axis
```

```
## Warning: Transformation introduced infinite values in continuous y-axis
```

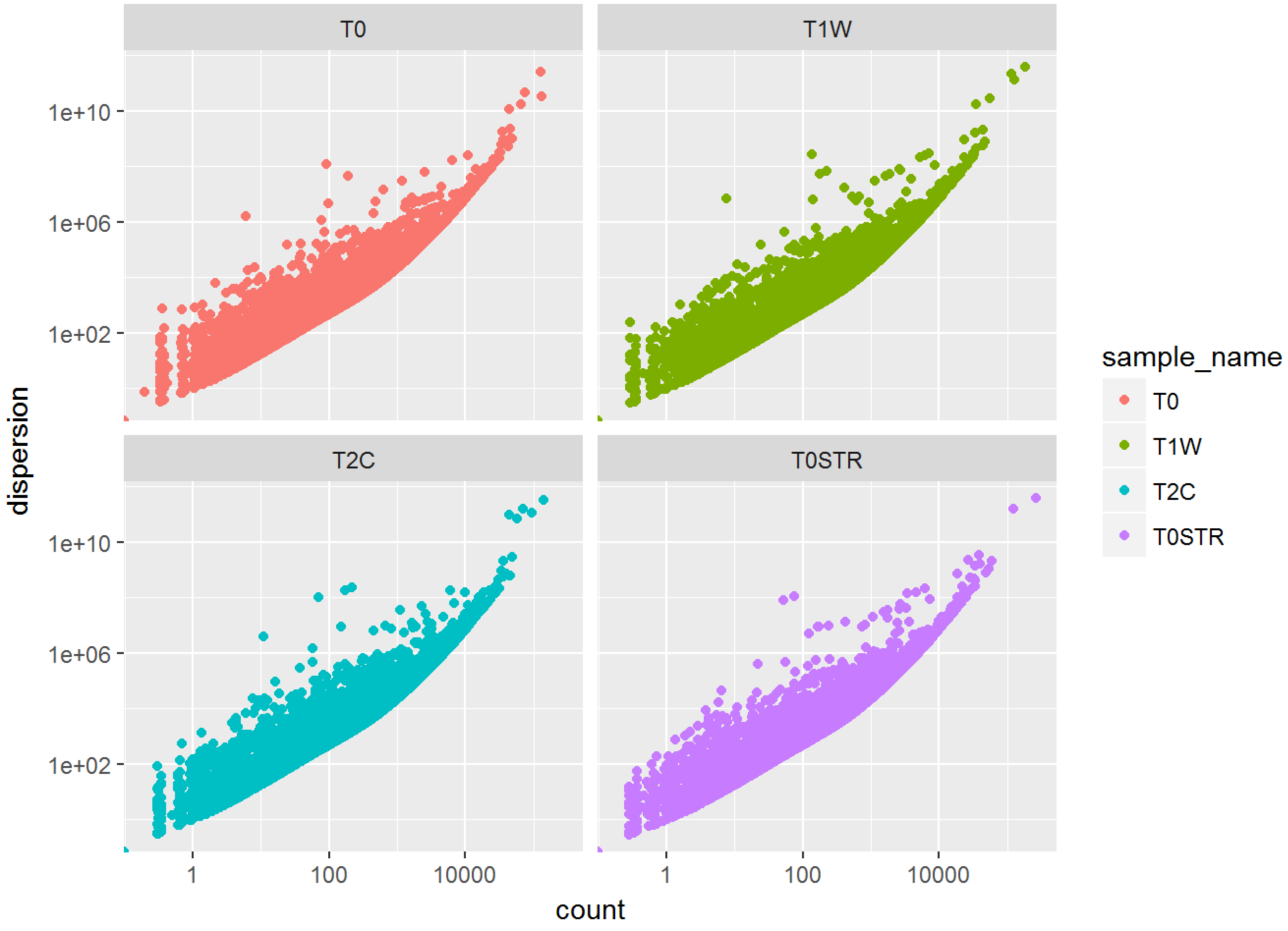


```
genes.scv <- fpkmSCVPlot(genes(cuff))
```

```
## Scale for 'x' is already present. Adding another scale for 'x', which
## will replace the existing scale.
```

```
genes.scv
```

```
## `geom_smooth()` using method = 'gam'
```

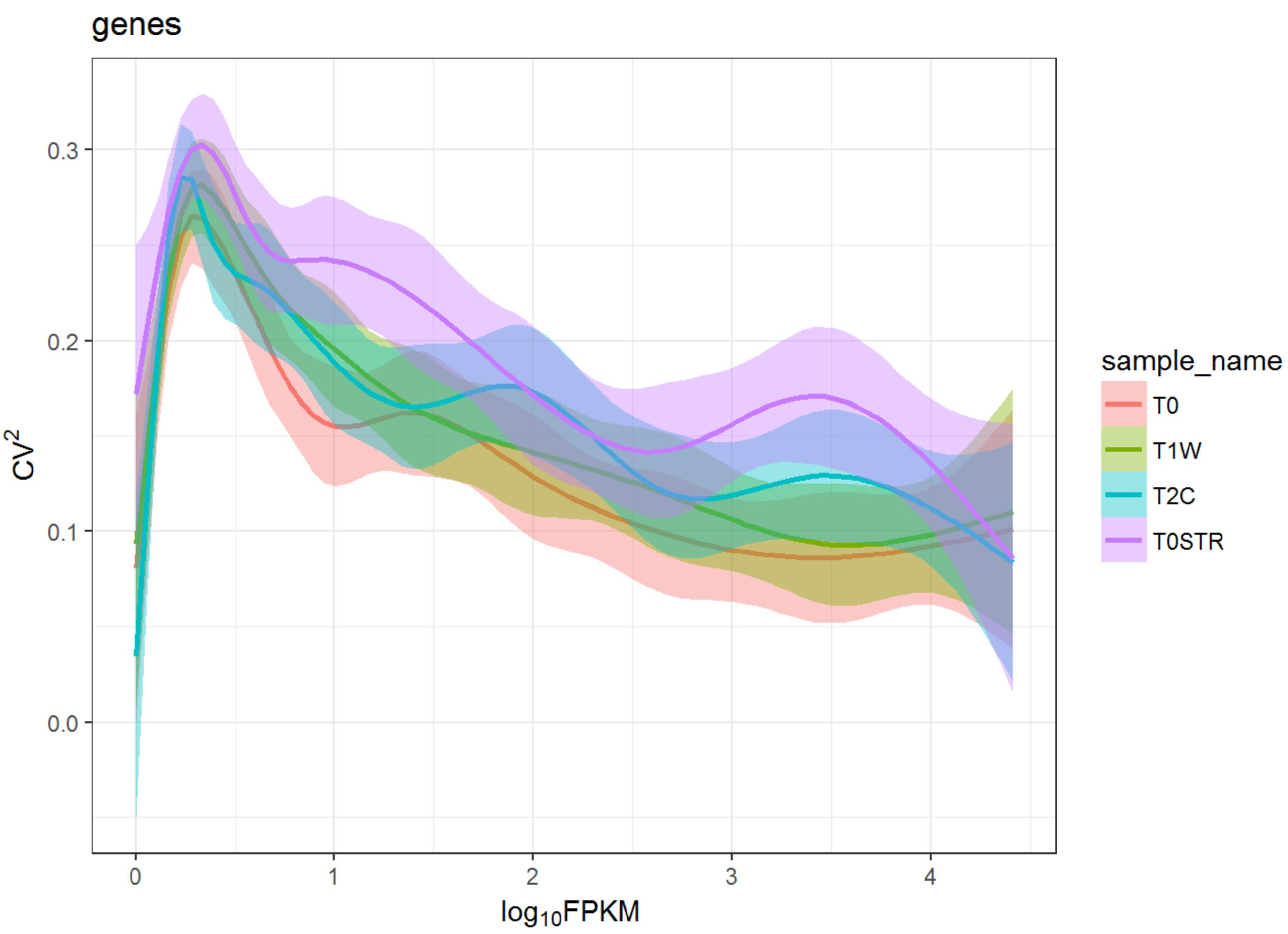


```
isoforms.scv <- fpkmSCVPlot(isoforms(cuff))
```

```
## Scale for 'x' is already present. Adding another scale for 'x', which
## will replace the existing scale.
```

```
isoforms.scv
```

```
## `geom_smooth()` using method = 'gam'
```

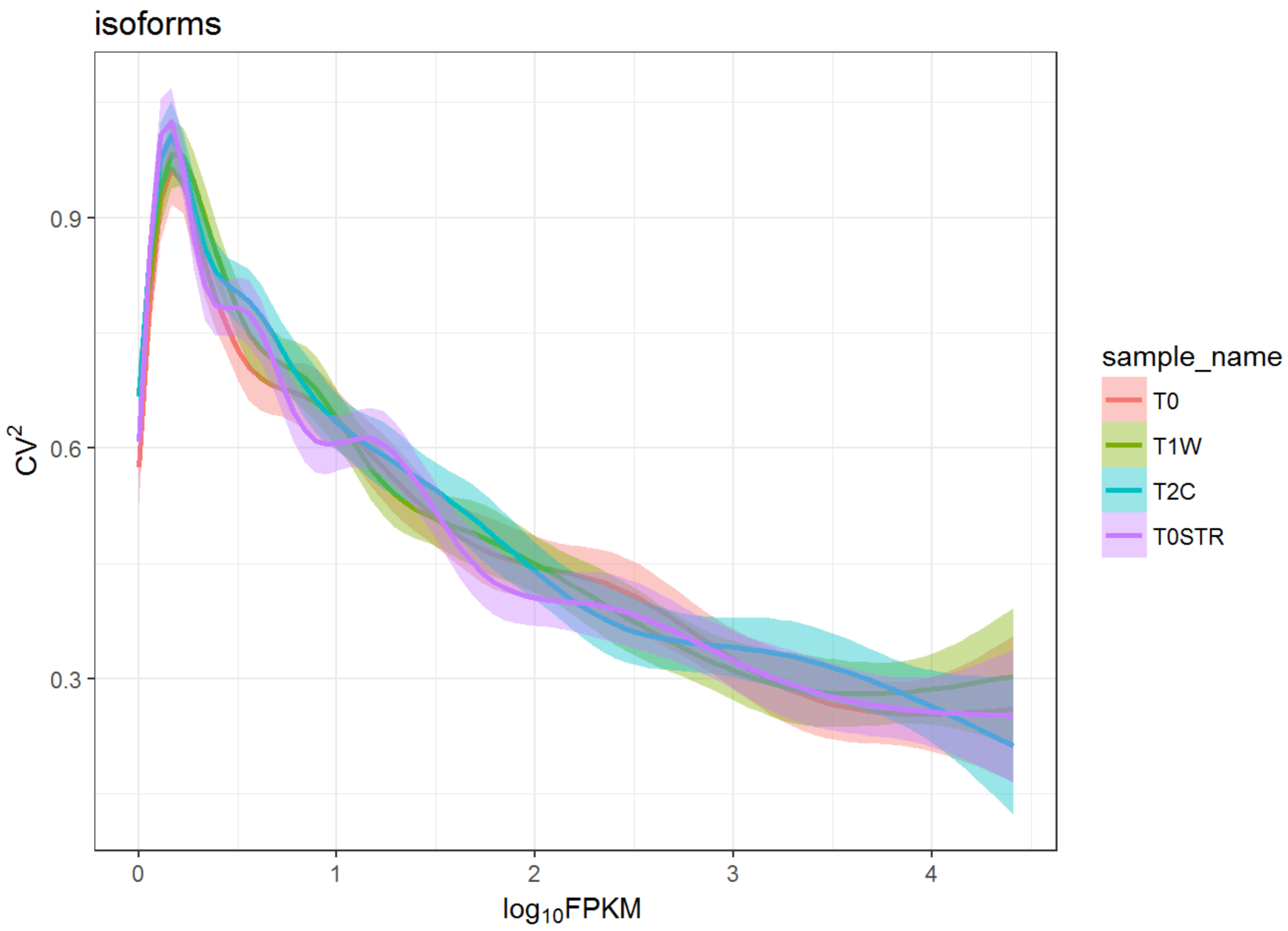


```
dens <- csDensity(genes(cuff))
dens
```

```
## Warning: Removed 2700 rows containing non-finite values (stat_density).
```

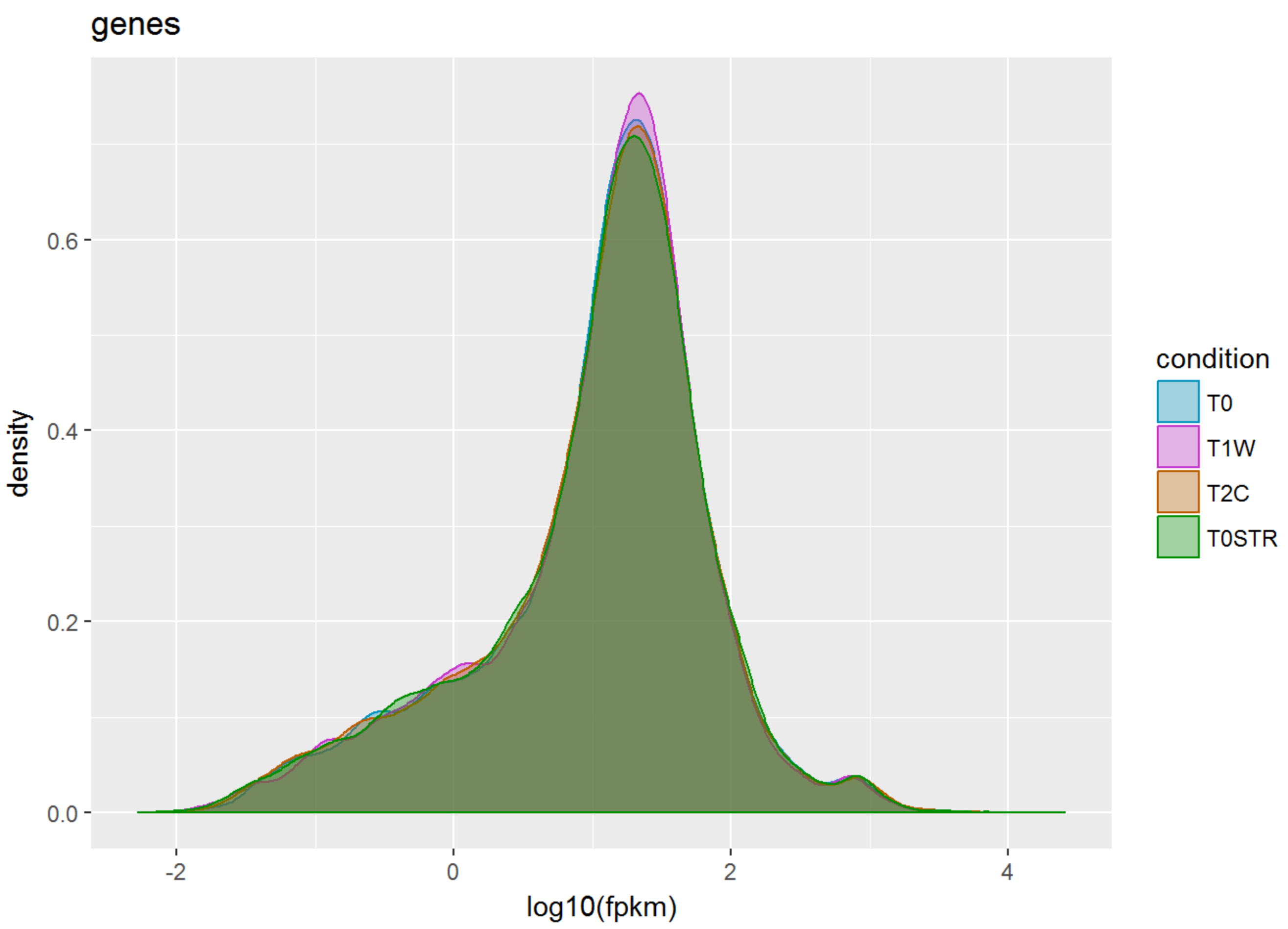


```
scat <- csScatterMatrix(genes(cuff))
scat
```

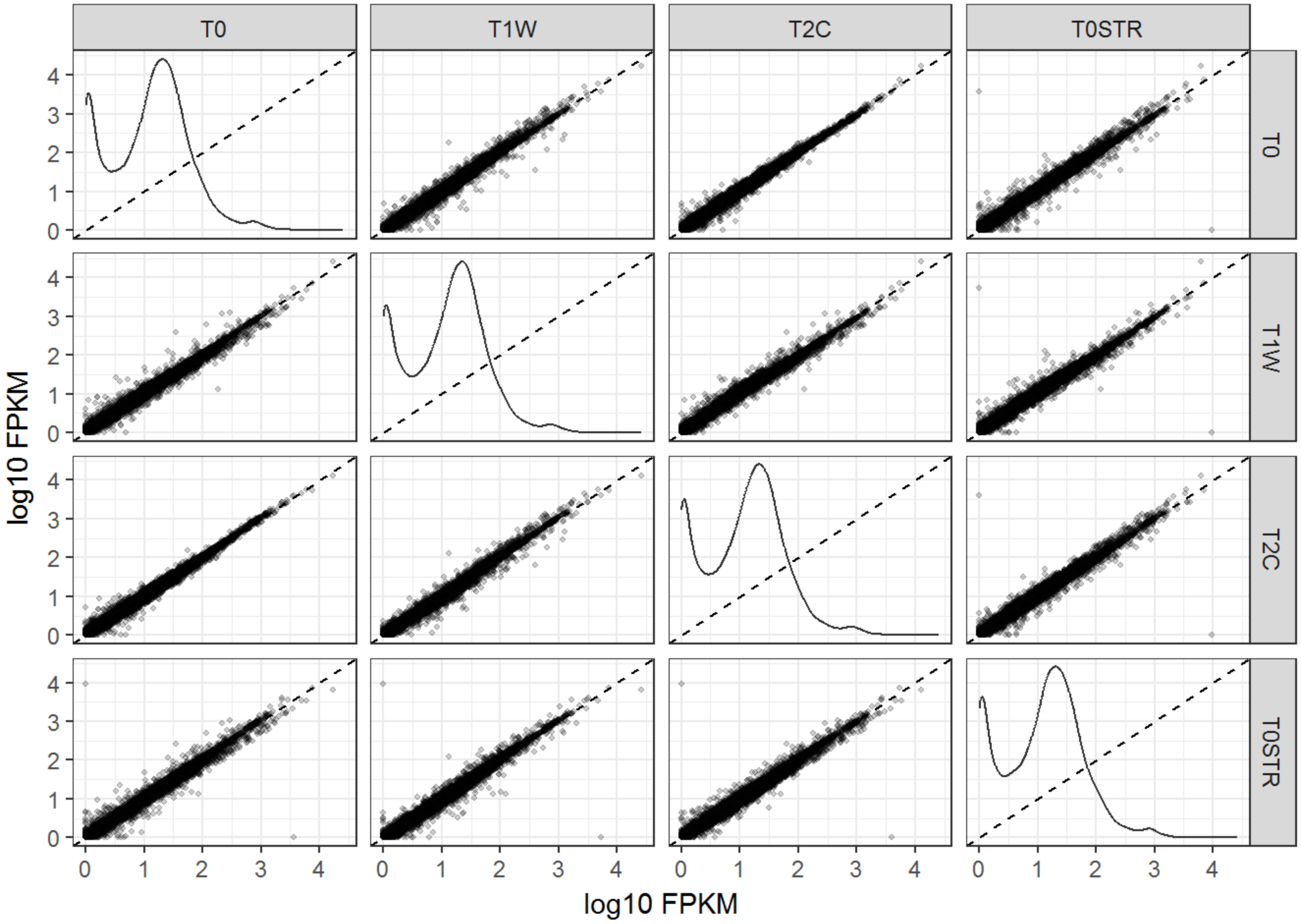


```
scat1 <- csScatter(genes(cuff), "T0", "T1W", smooth = TRUE)
scat1
```

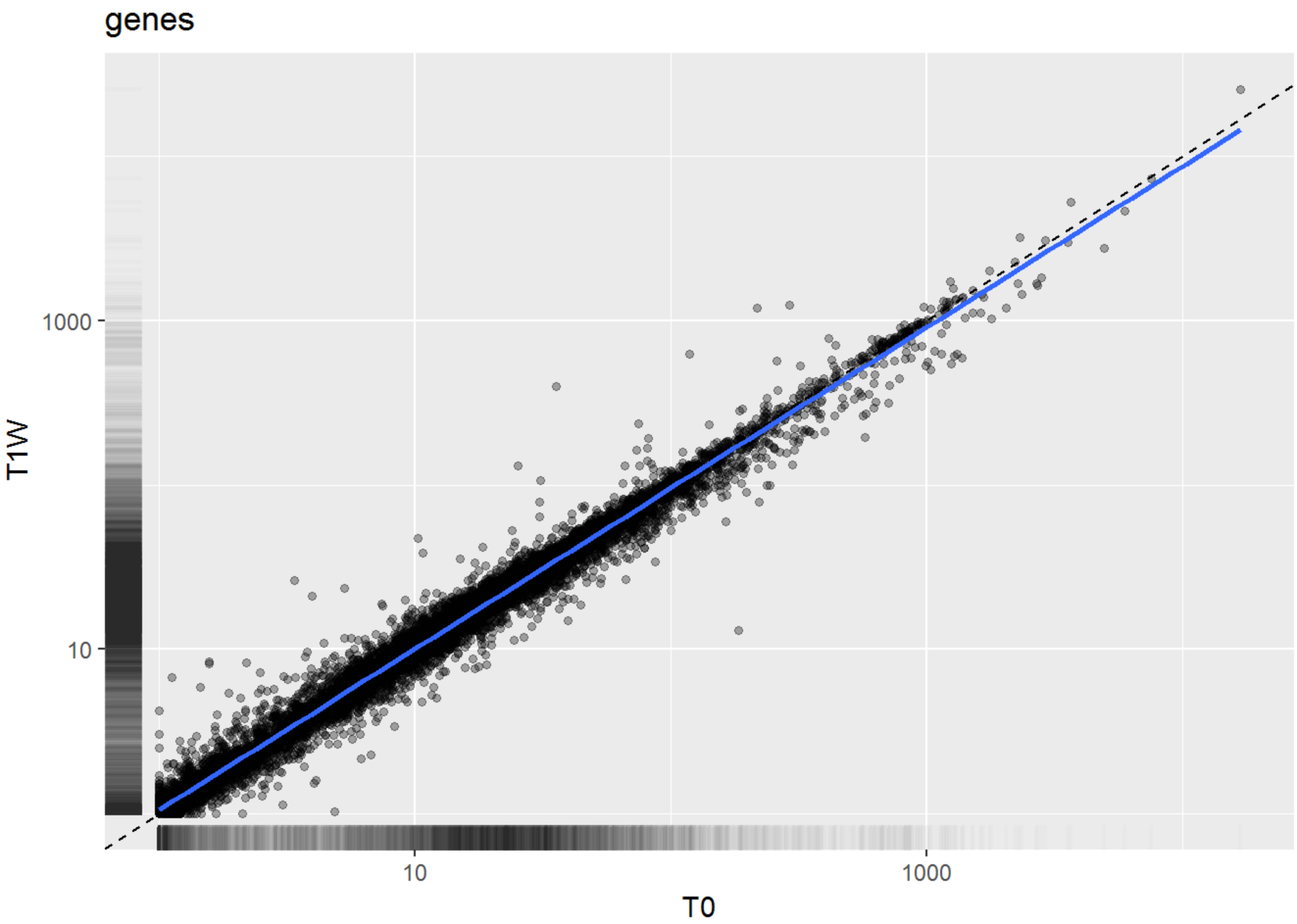


```
scat2 <- csScatter(genes(cuff), "T0", "T2C", smooth = TRUE)
scat2
```

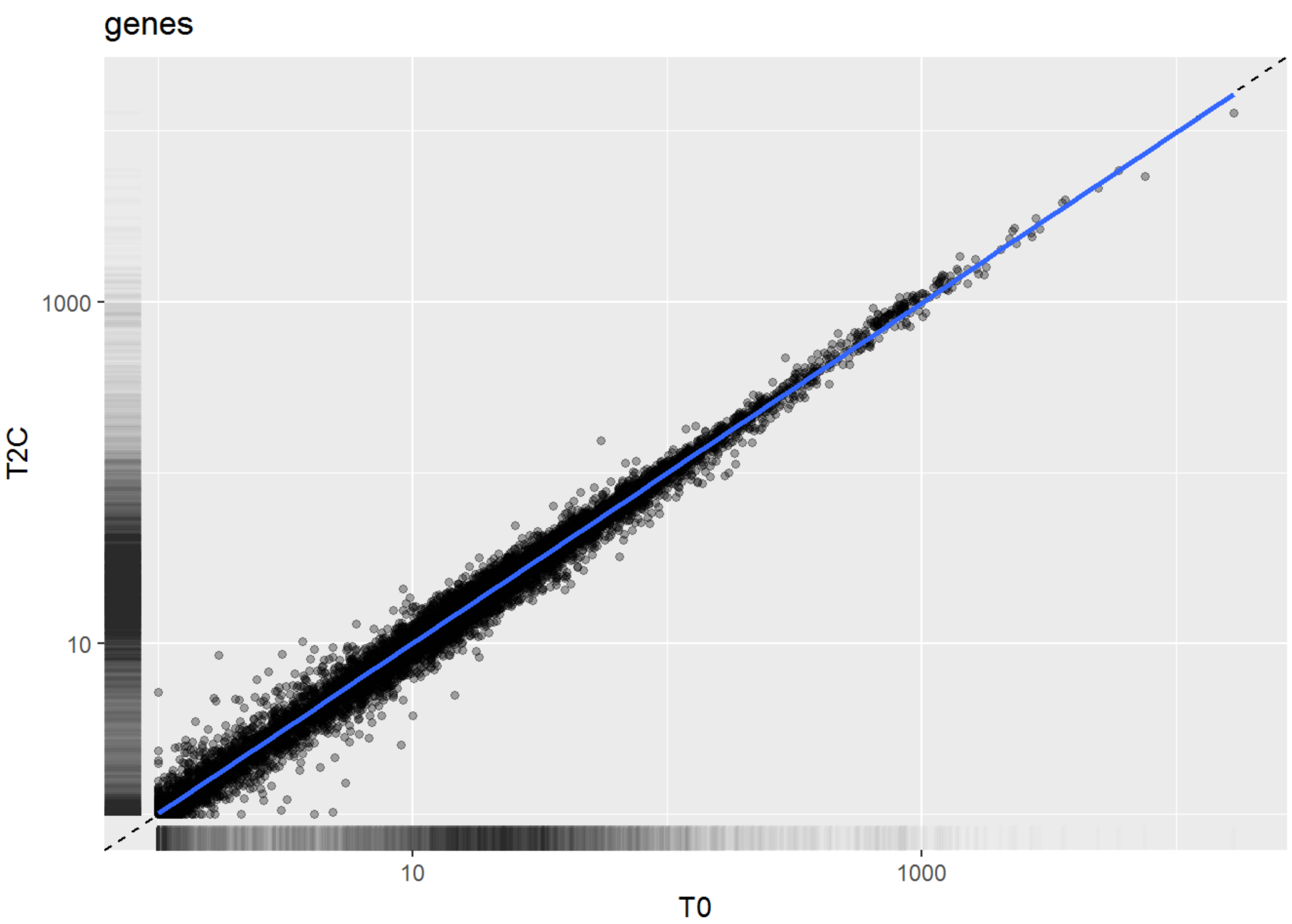


```
scat3 <- csScatter(genes(cuff), "T0", "T0STR", smooth = TRUE)
scat3
```

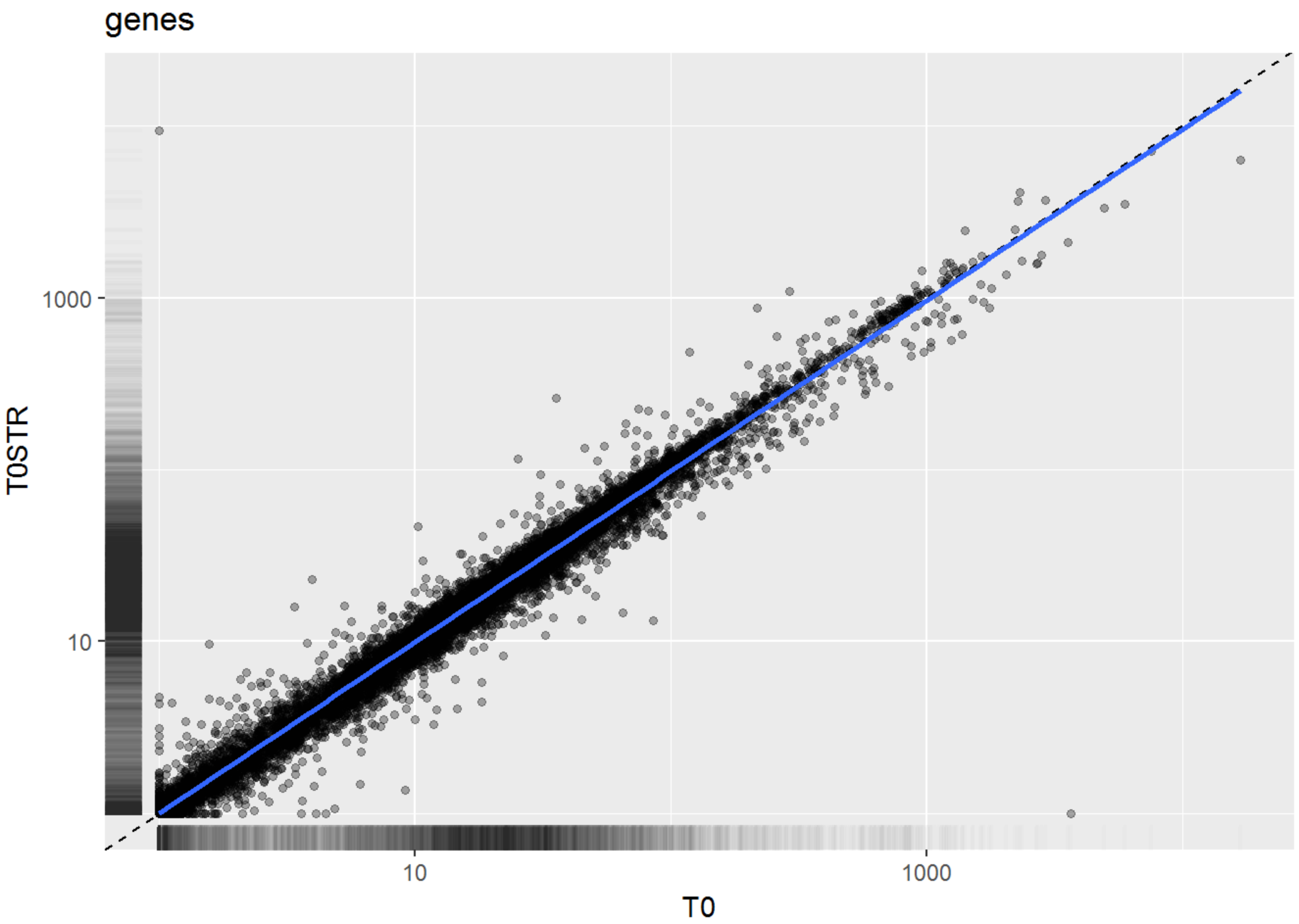


```
scat4 <- csScatter(genes(cuff), "T1W", "T2C", smooth = TRUE)
scat4
```

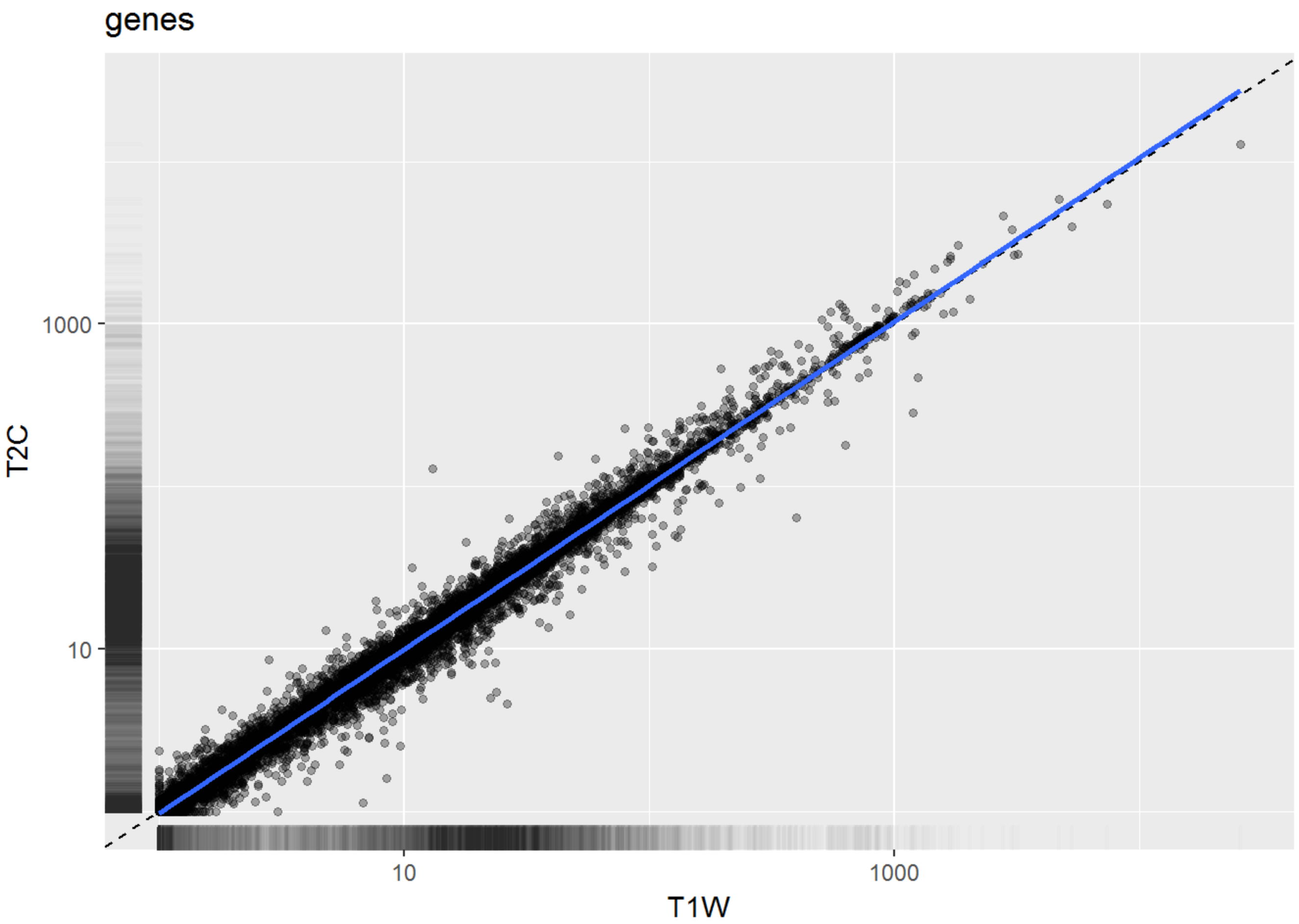


```
scat5 <- csScatter(genes(cuff), "T1W", "T0STR", smooth = TRUE)
scat5
```

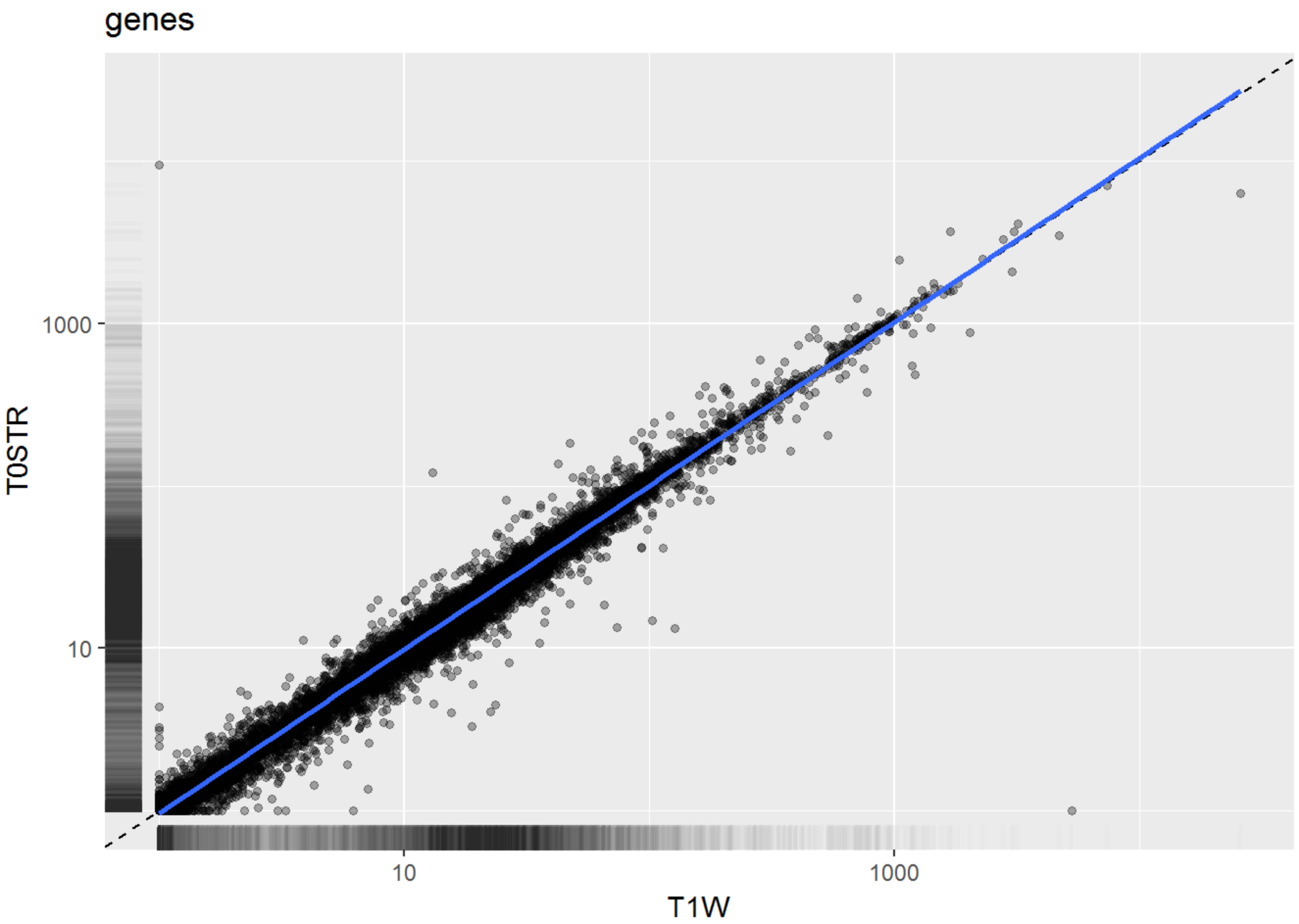


```
scat6 <- csScatter(genes(cuff), "T2C", "T0STR", smooth = TRUE)
scat6
```

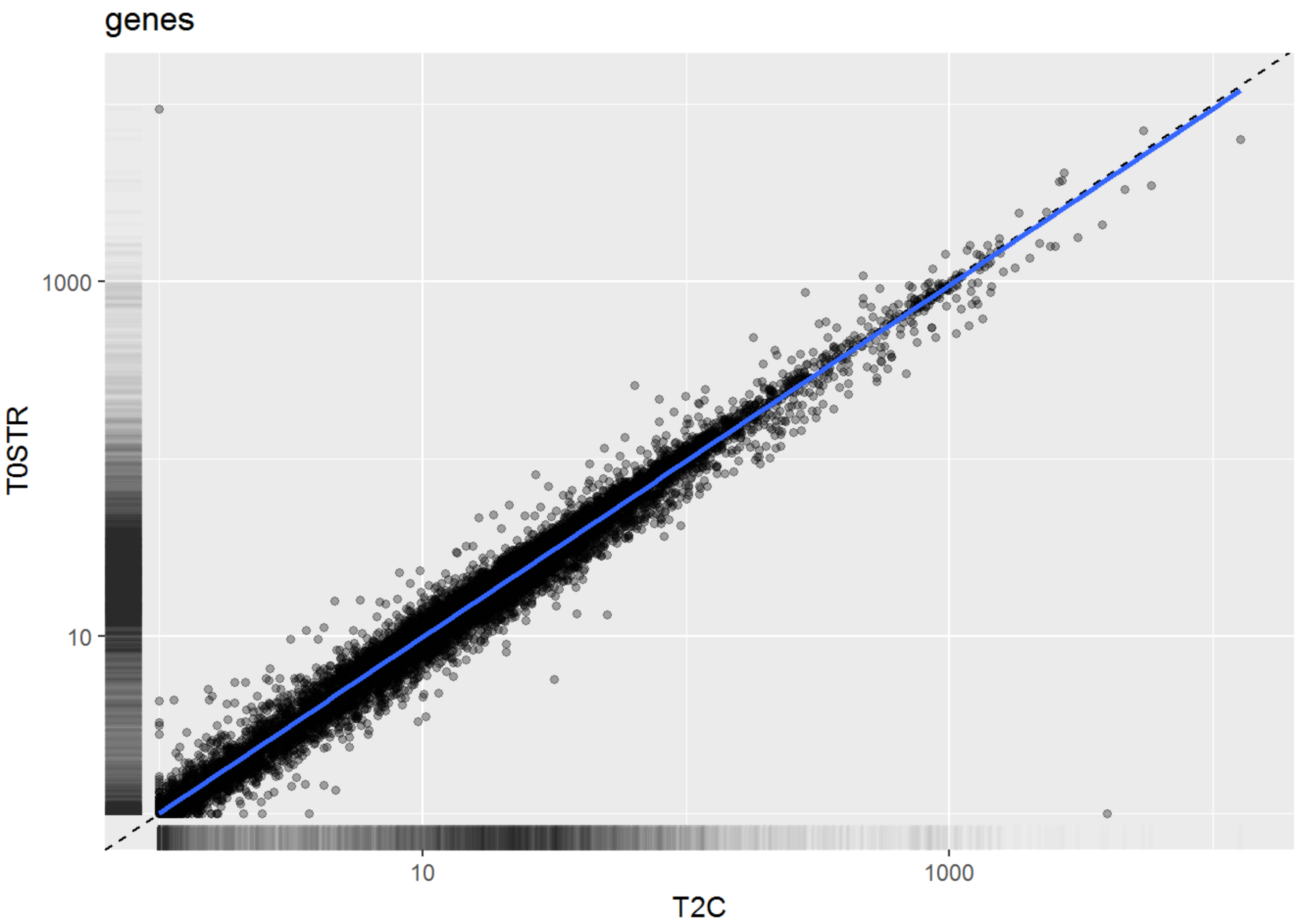


```
dend <- csDendro(genes(cuff))
```

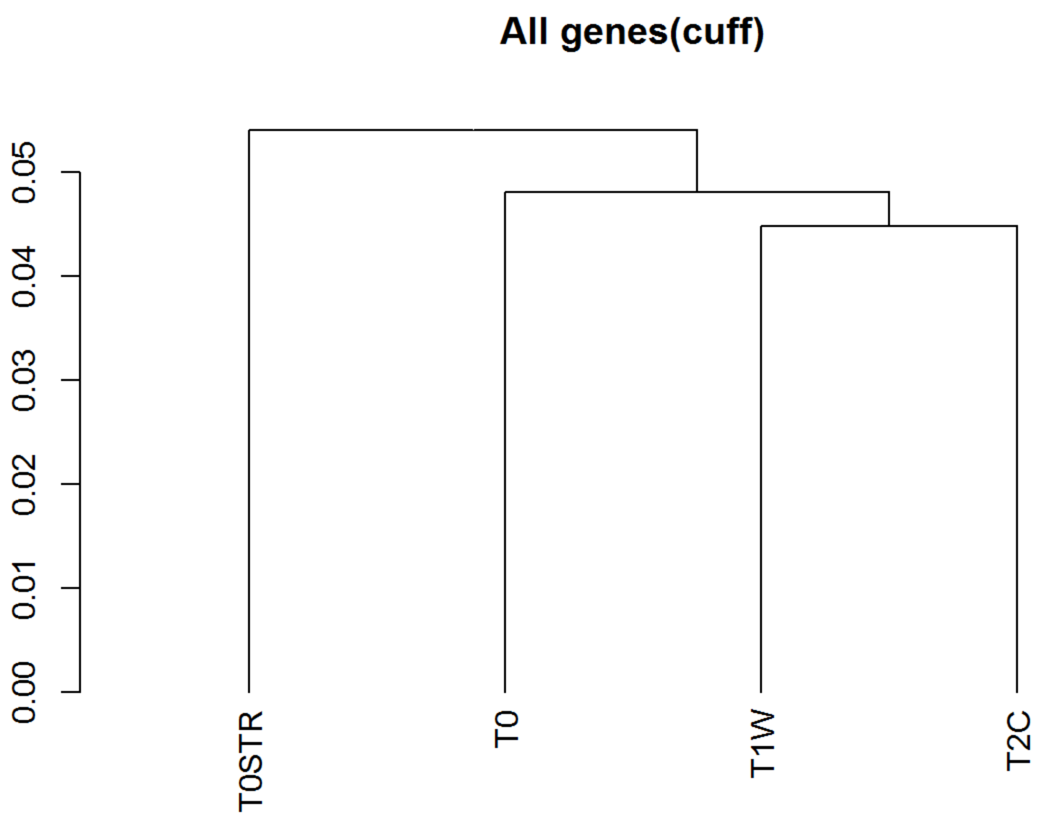


```
dend
```

```
## 'dendrogram' with 2 branches and 4 members total, at height 0.05399459
```

```
volc <- csVolcanoMatrix(genes(cuff))
volc
```

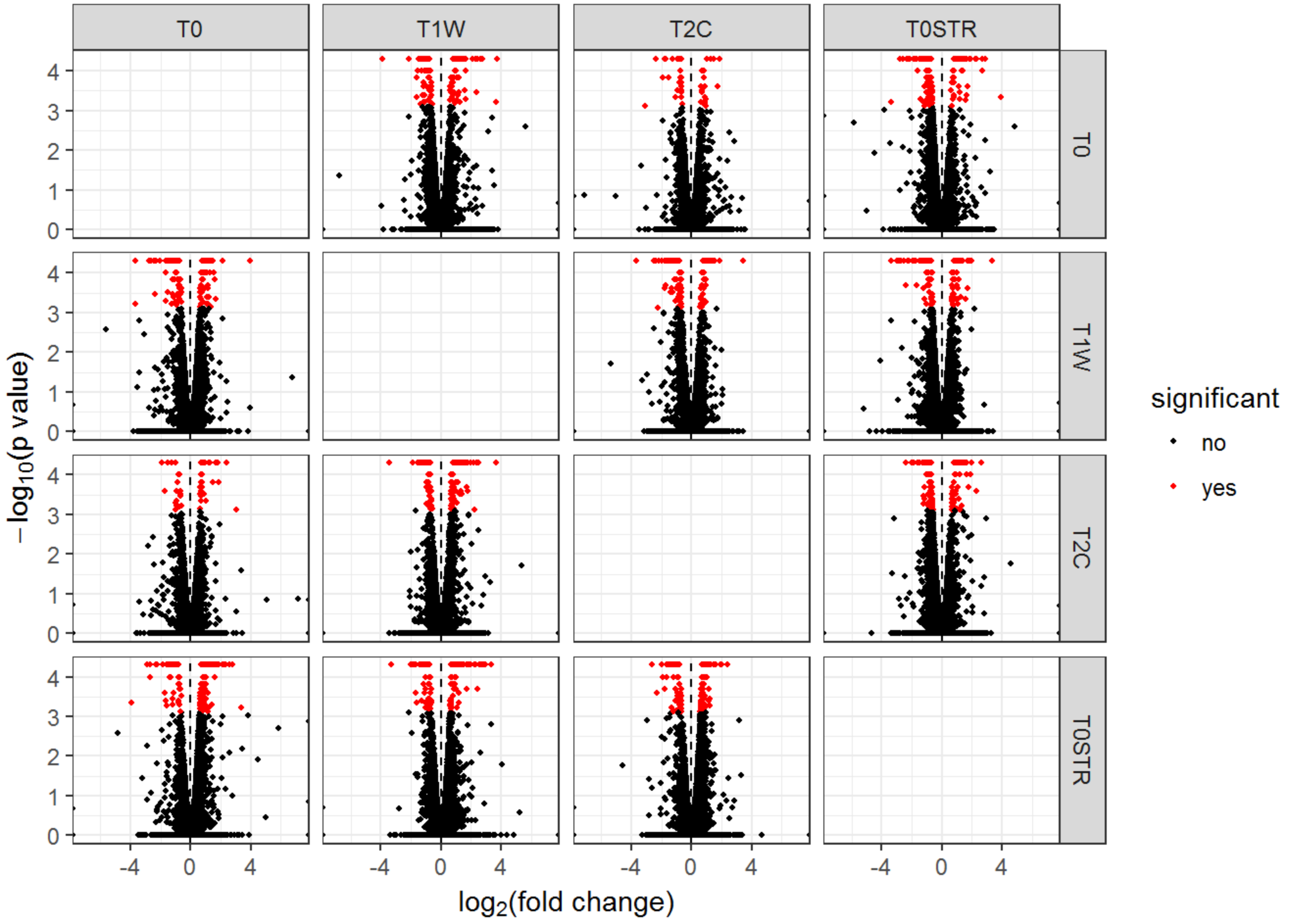


```
volc1 <- csVolcano(genes(cuff), "T0", "T1W", smooth = TRUE, showSignificant = TRUE,
alpha = 0.05)
volc1
```

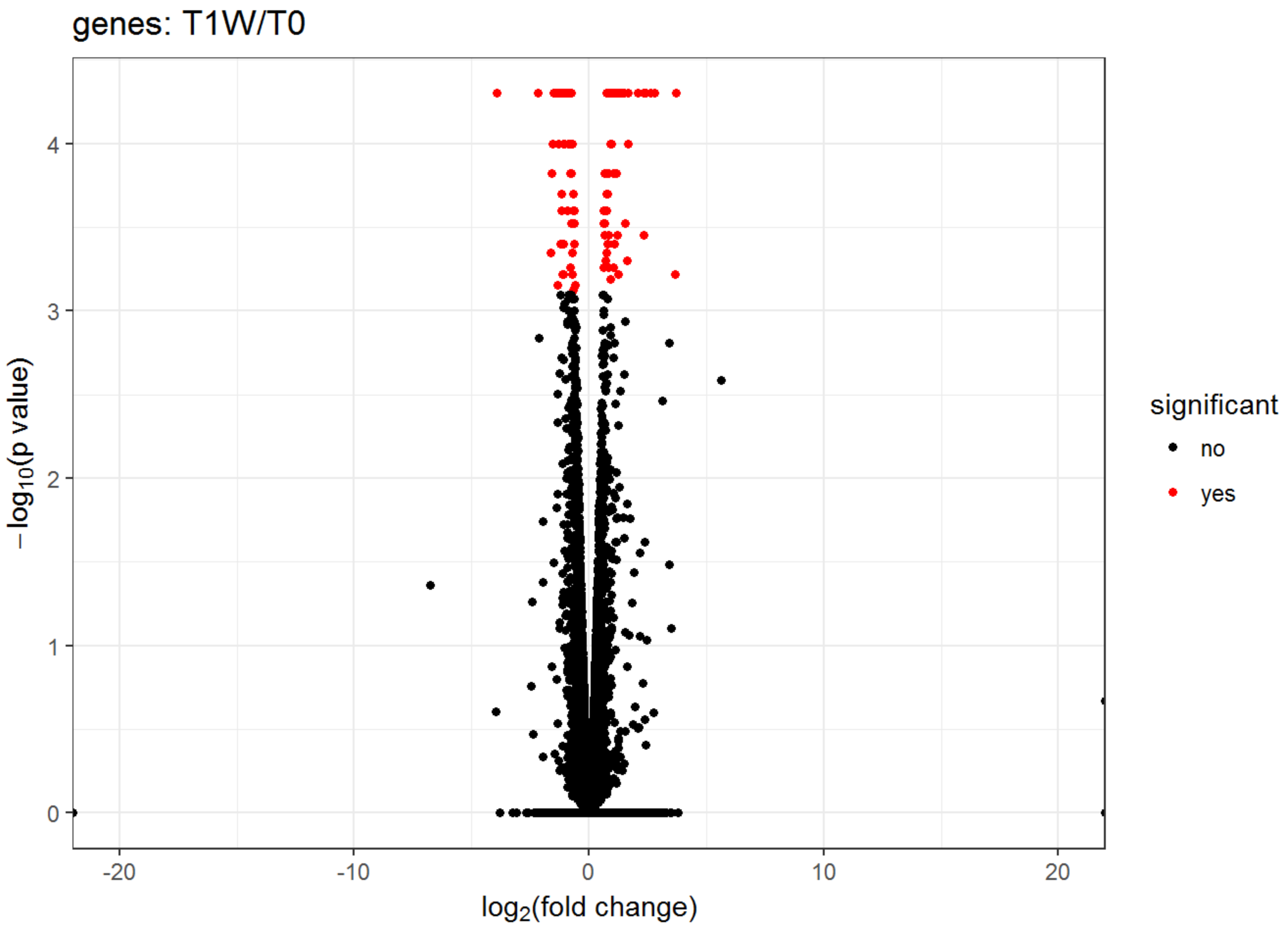


```
volc2 <- csVolcano(genes(cuff), "T0", "T2C", smooth = TRUE, showSignificant = TRUE,
alpha = 0.05)
volc2
```

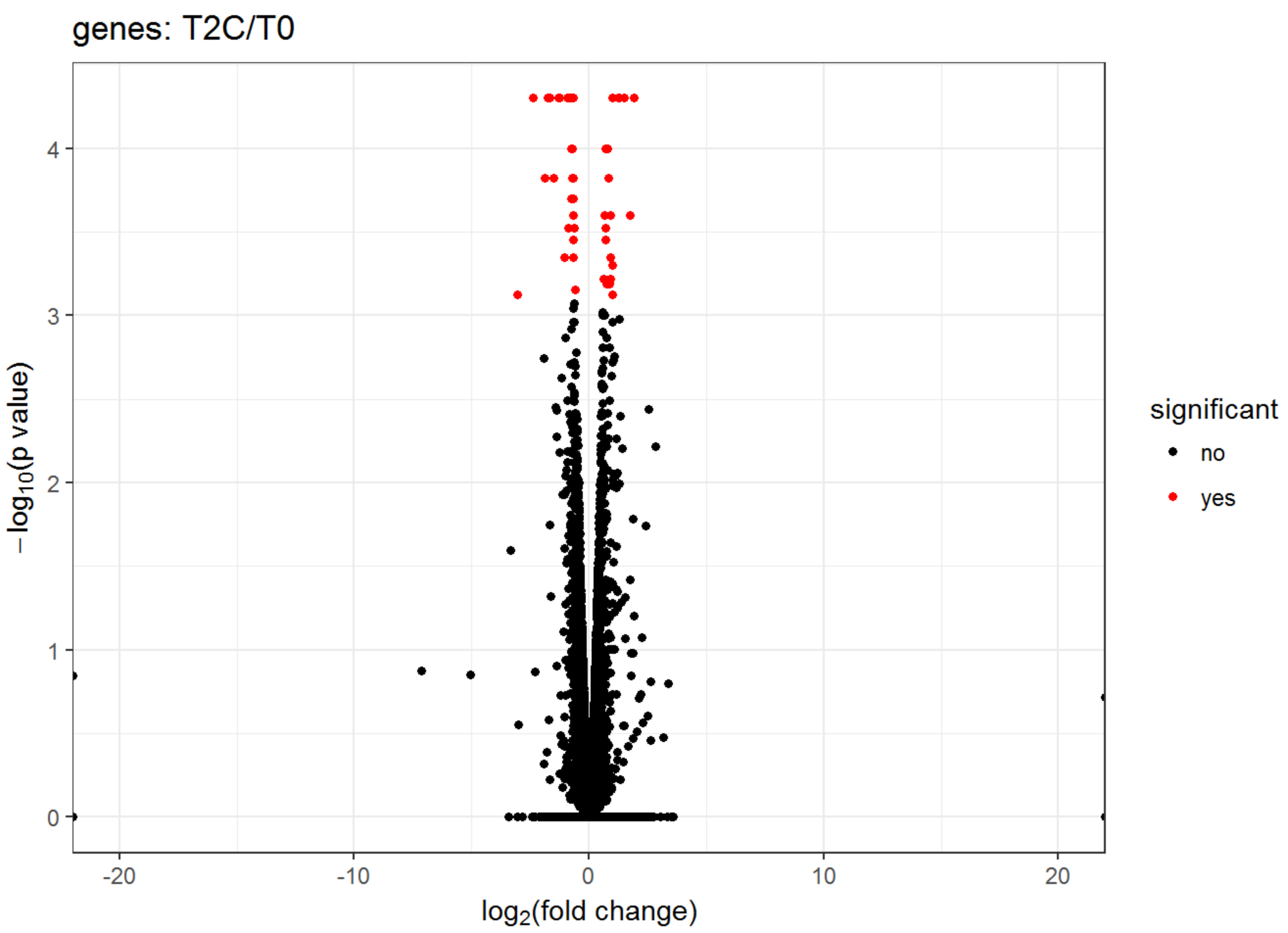


```
volc3 <- csVolcano(genes(cuff), "T0", "T0STR", smooth = TRUE, showSignificant = TRU
E, alpha = 0.05)
volc3
```

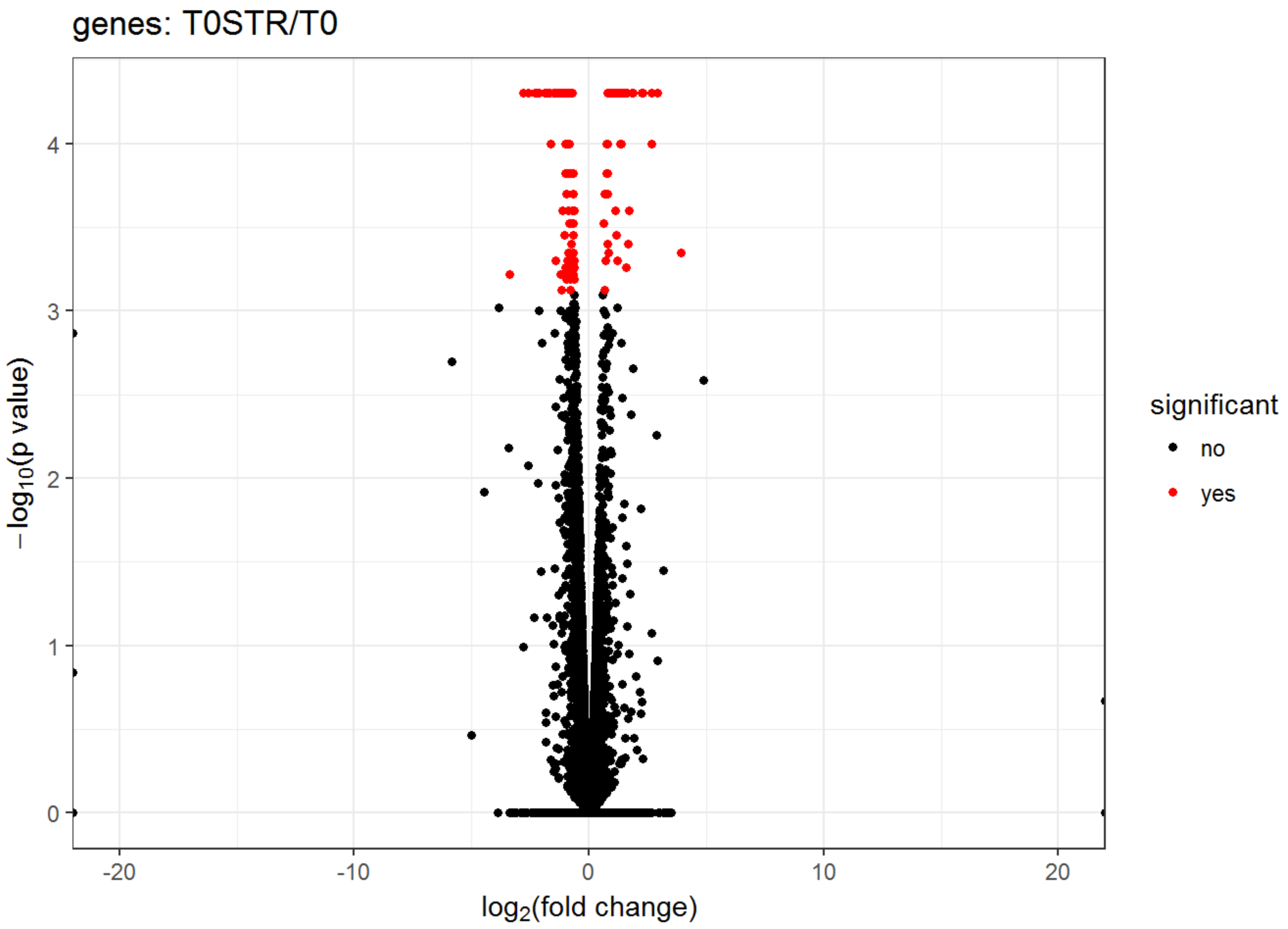


```
volc4 <- csVolcano(genes(cuff), "T1W", "T2C", smooth = TRUE, showSignificant = TRUE
, alpha = 0.05)
volc4
```

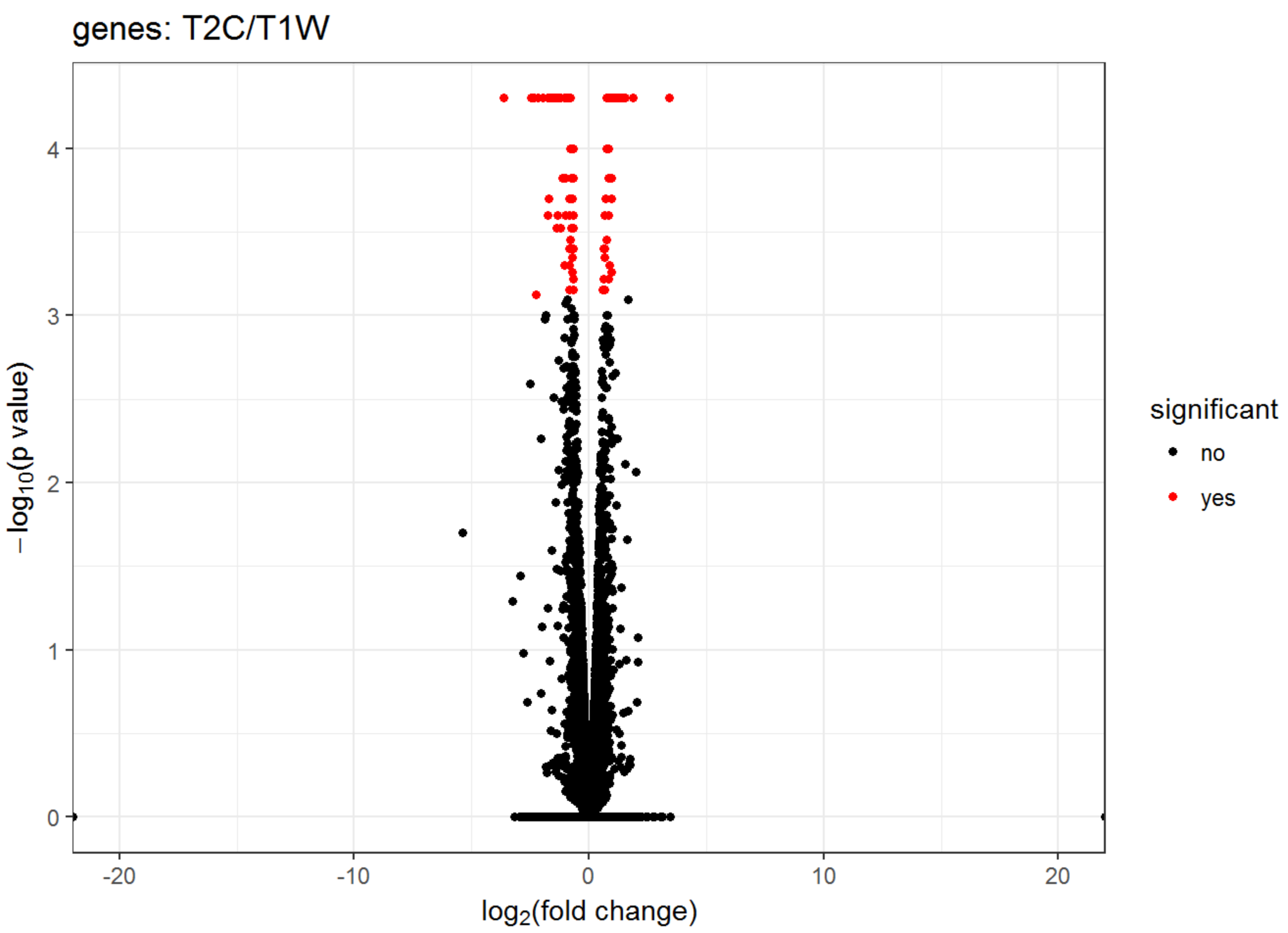


```
volc5 <- csVolcano(genes(cuff), "T1W", "T0STR", smooth = TRUE, showSignificant = TR
UE, alpha = 0.05)
volc5
```

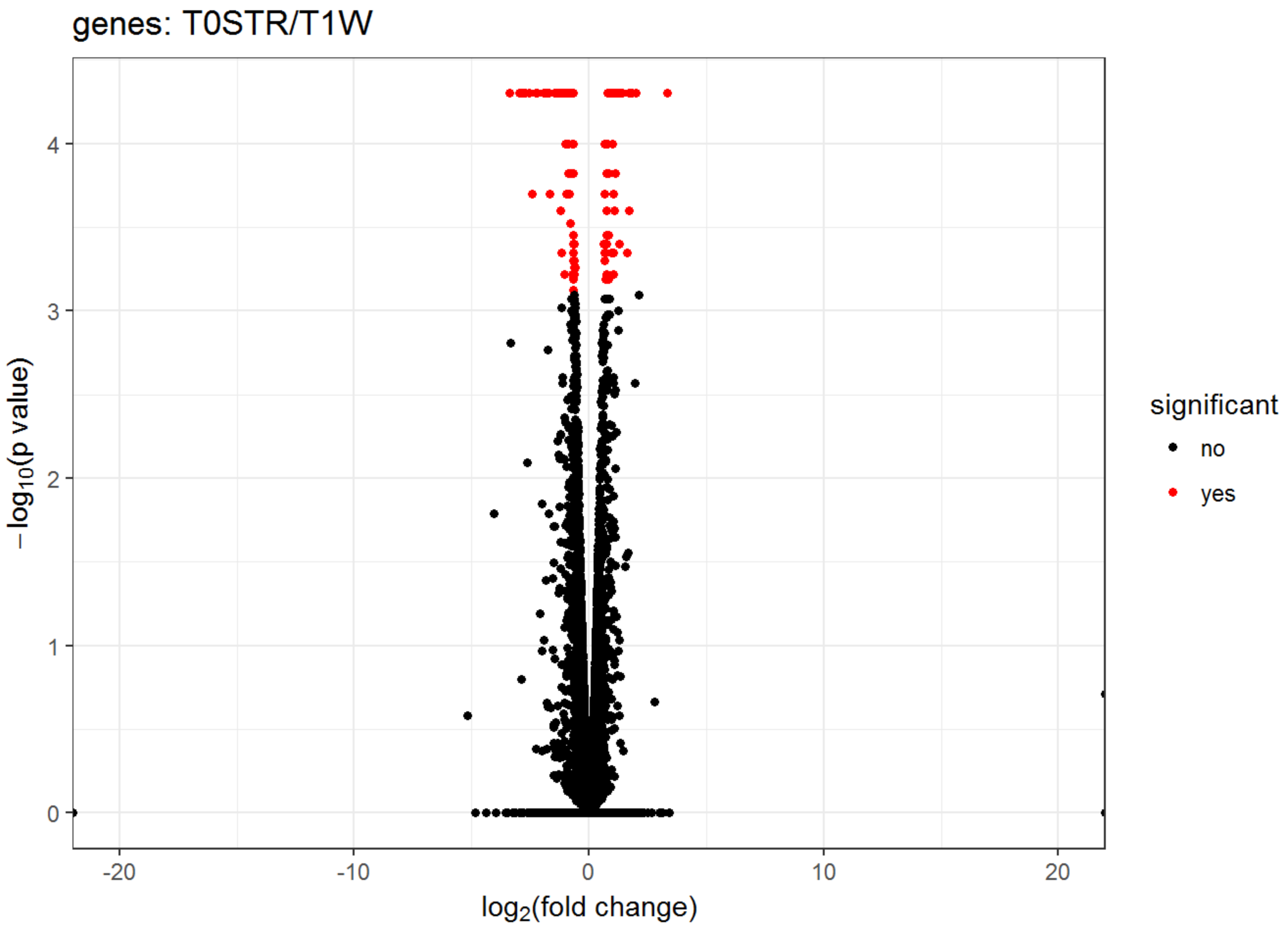


```
volc6 <- csVolcano(genes(cuff), "T2C", "T0STR", smooth = TRUE, showSignificant = TR
UE, alpha = 0.05)
volc6
```

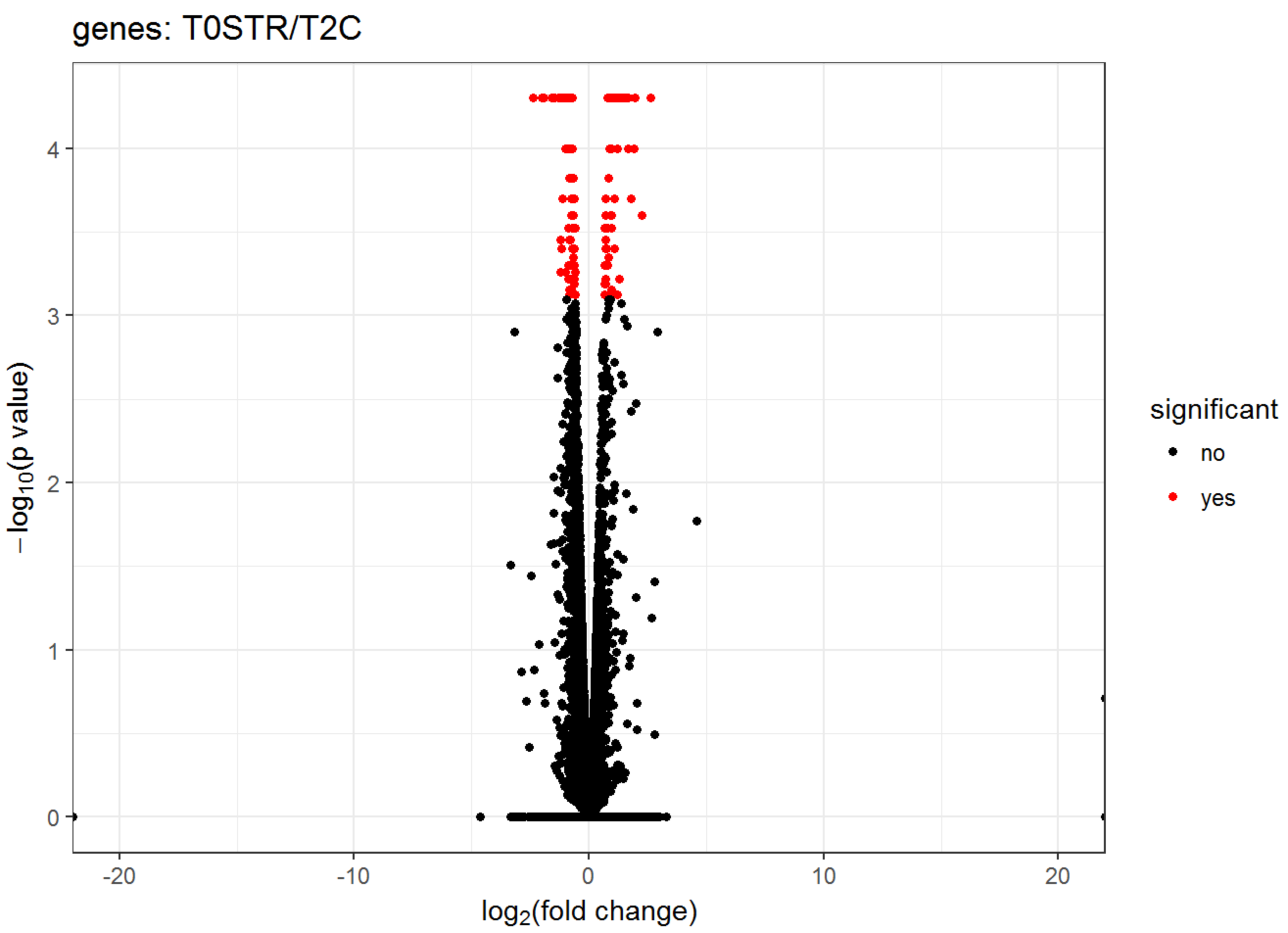

Annoying way to pull all gene IDs, set alpha to 1:

```
allGeneIDs <- getSig(cuff, alpha = 1, level = "genes")
allGenes <- getGenes(cuff, allGeneIDs)
```

```
## Warning: Closing open result set, pending rows

## Warning: Closing open result set, pending rows
```

```
sigmat <- sigMatrix(cuff, level = "genes", alpha = 0.05)
```

```
## Warning: `show_guide` has been deprecated. Please use `show.legend`
## instead.
```

```
sigmat
```

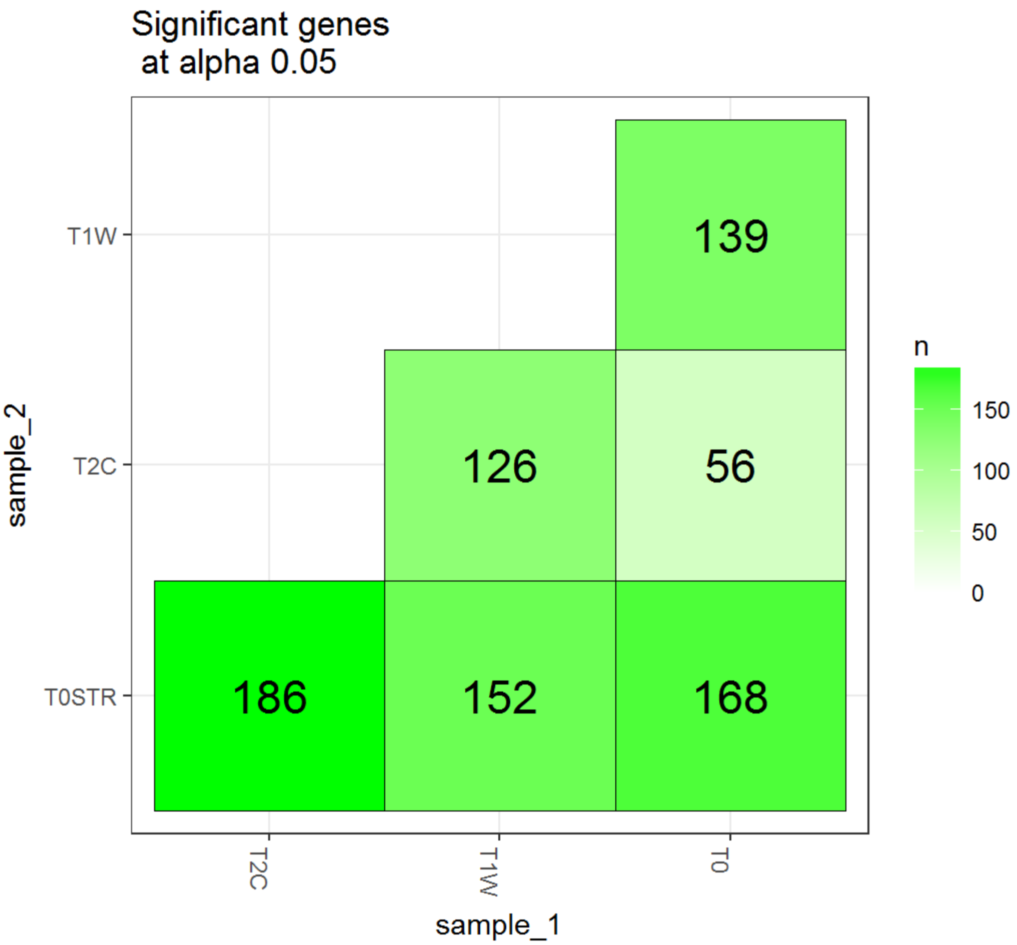

Get significant genes:

```
sigGeneIDs <- getSig(cuff, alpha = 0.05, level = "genes")
sigGenes <- getGenes(cuff, sigGeneIDs)
```

```
## Warning: Closing open result set, pending rows

## Warning: Closing open result set, pending rows
```

```
heat <- csHeatmap(sigGenes, cluster = 'both')
```

```
## Using tracking_id, sample_name as id variables
```

```
## No id variables; using all as measure variables
```

```
heat
```

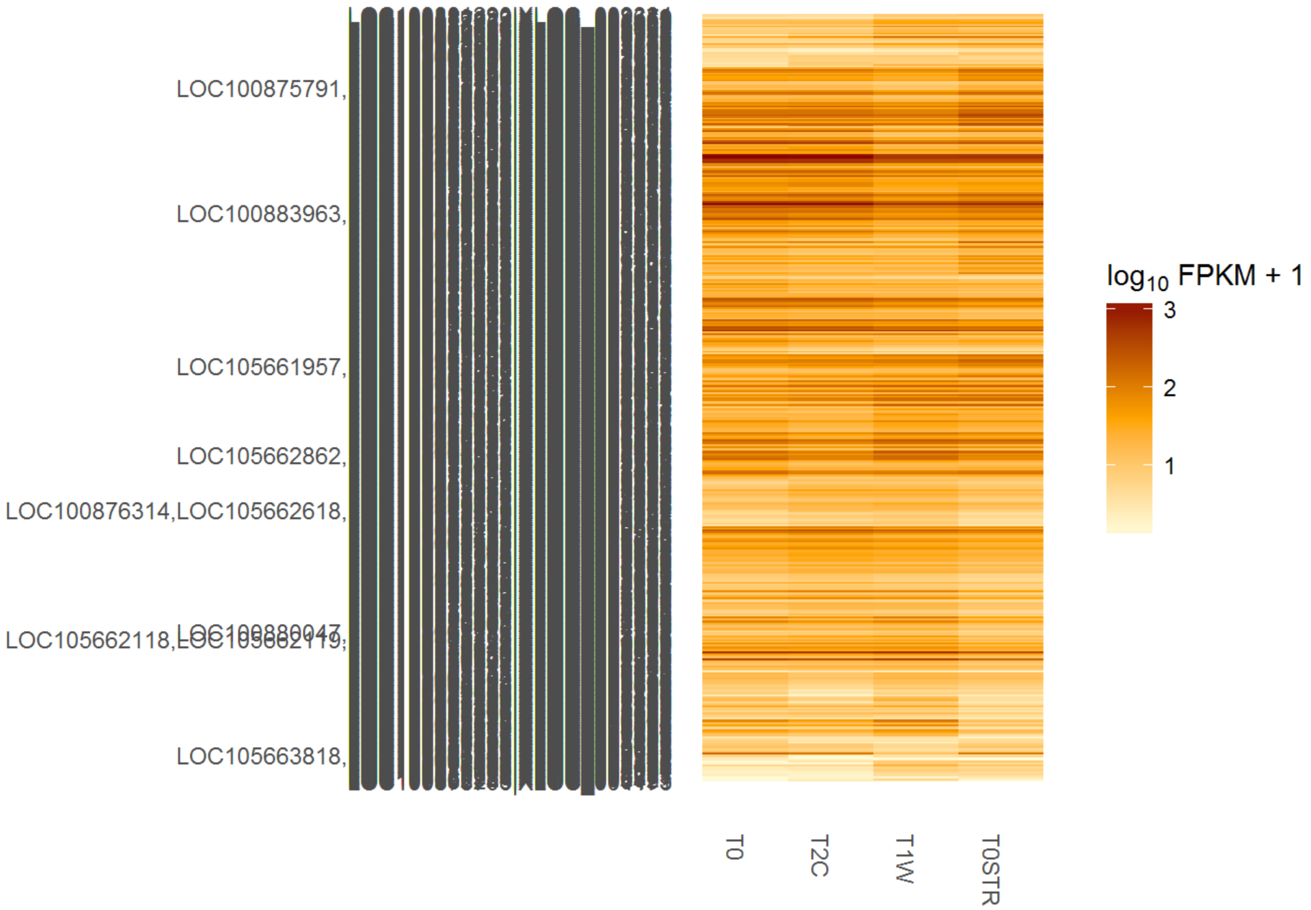


```
distHeat <- csDistHeat(genes(cuff))
distHeat
```

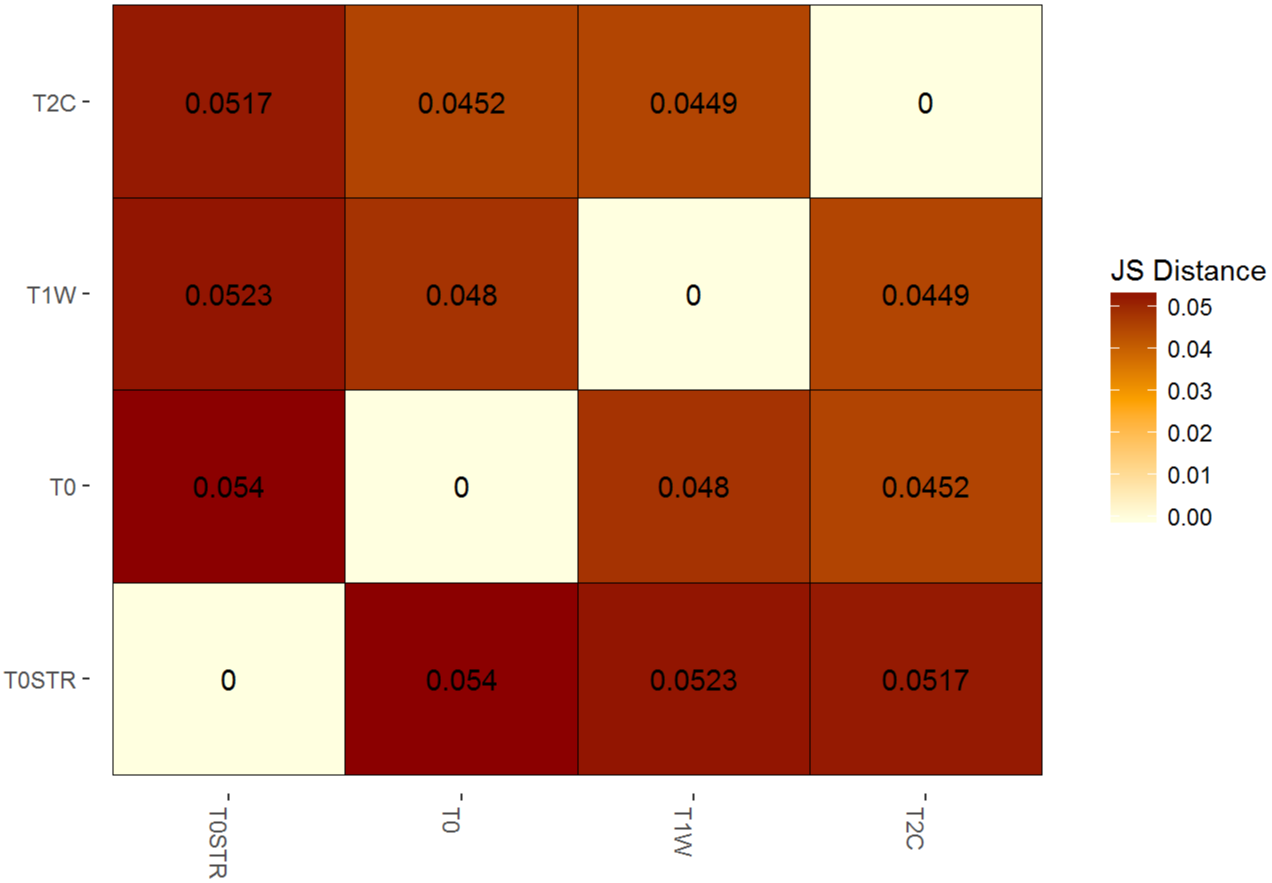

PCA plots:

```
genes.PCA <- PCAplot(genes(cuff))
```

```
## Warning: Ignoring unknown aesthetics: label
```

```
genes.PCA
```

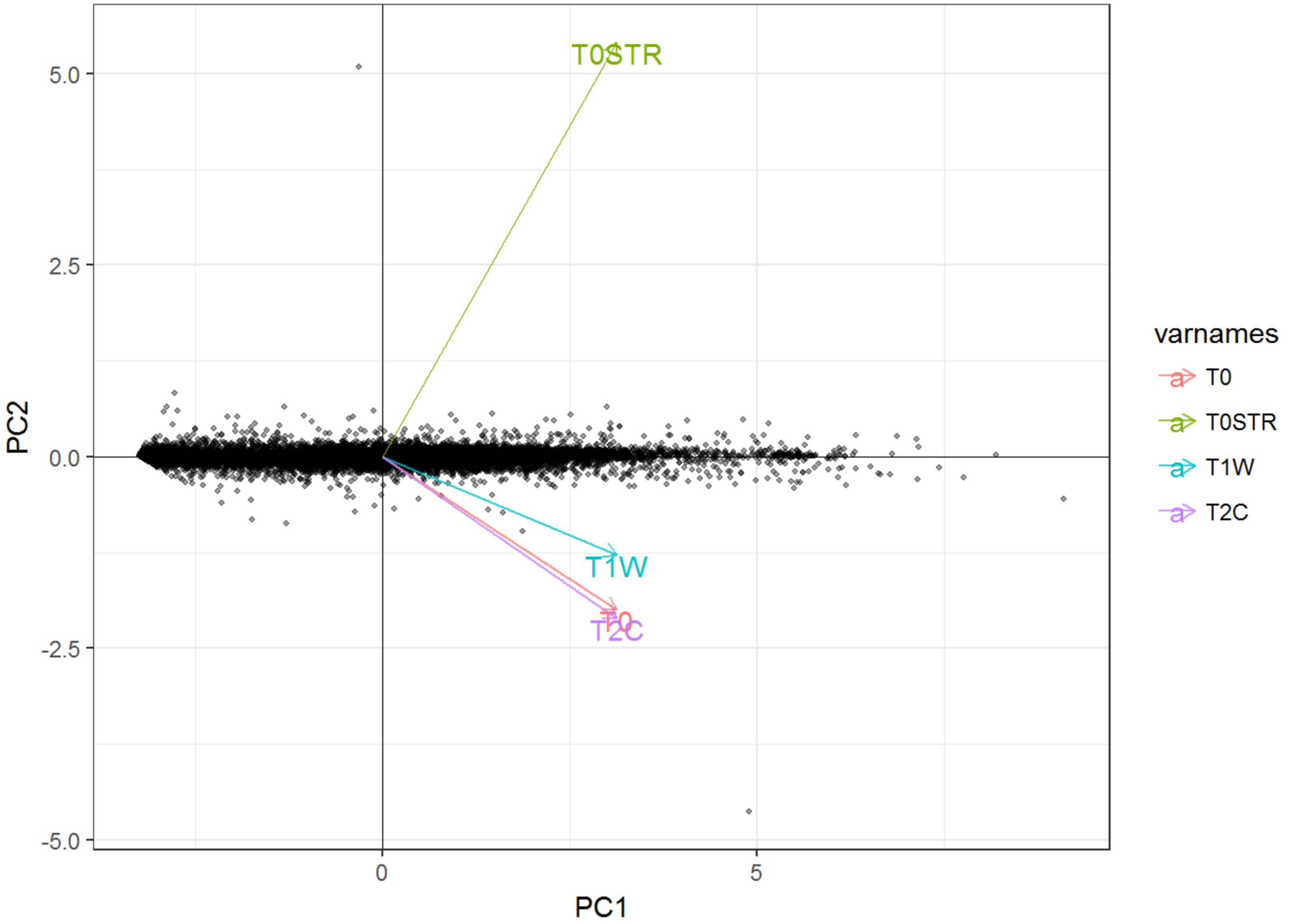


```
genes.PCA.reps <- PCAplot(genes(cuff), replicates = TRUE)
```

```
## Warning: Ignoring unknown aesthetics: label
```

```
genes.PCA.reps
```

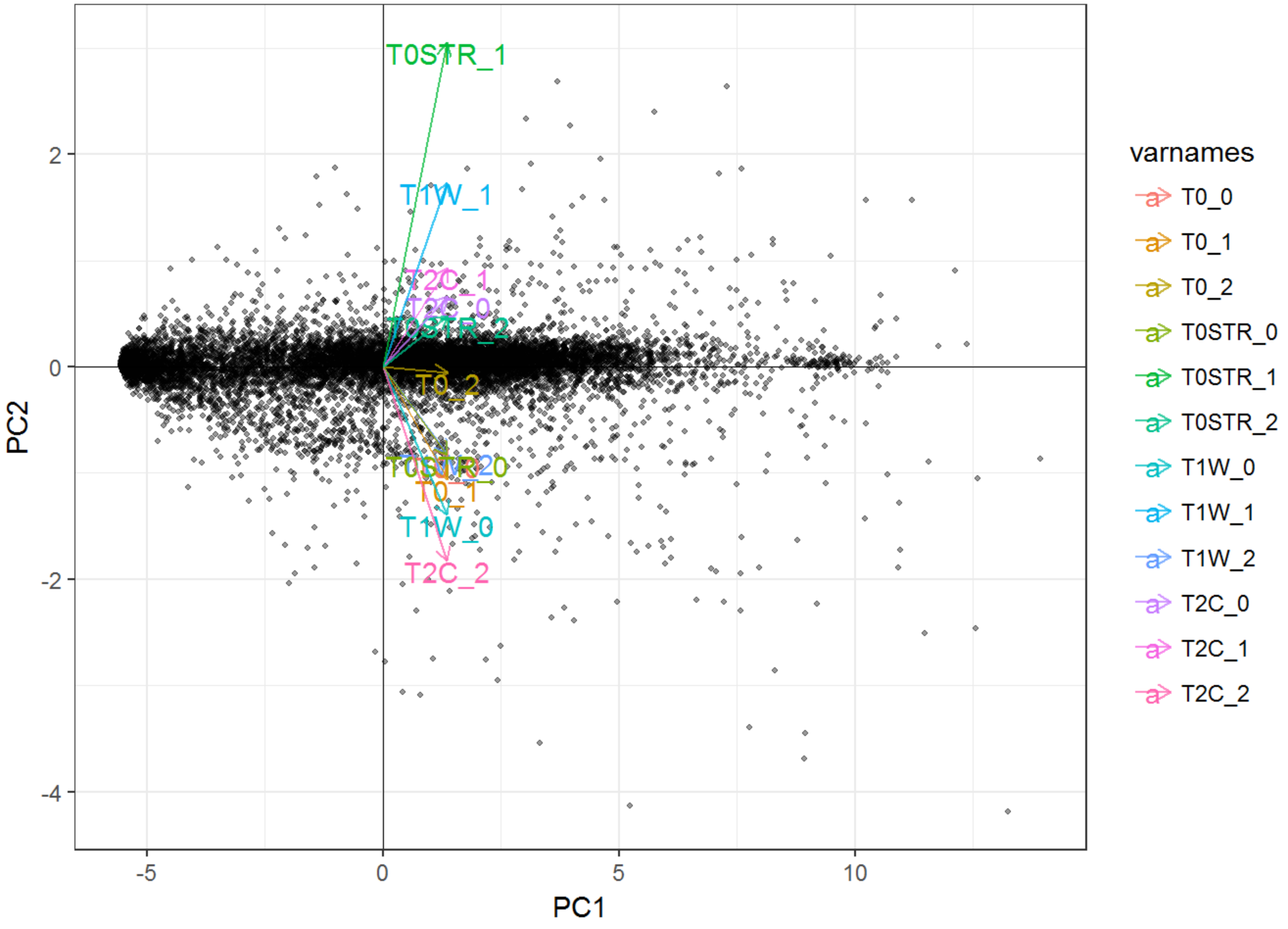


```
sig.PCA <- PCAplot(sigGenes)
```

```
## Using tracking_id, sample_name as id variables
```

```
## Warning: Ignoring unknown aesthetics: label
```

```
sig.PCA
```

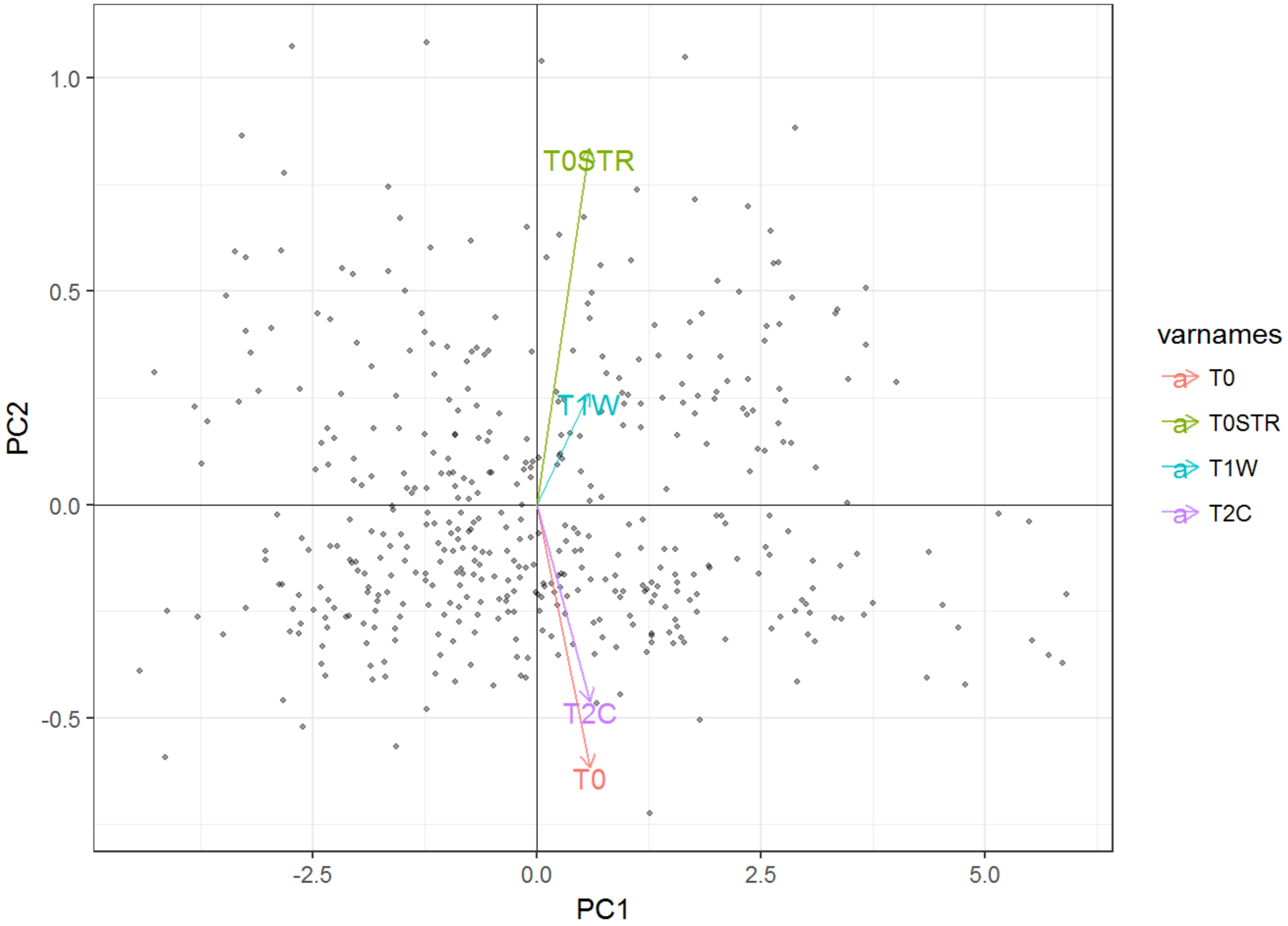


```
sig.PCA.reps <- PCAplot(sigGenes, replicates = TRUE)
```

```
## Using tracking_id, rep_name as id variables
```

```
## Warning: Ignoring unknown aesthetics: label
```

```
sig.PCA.reps
```

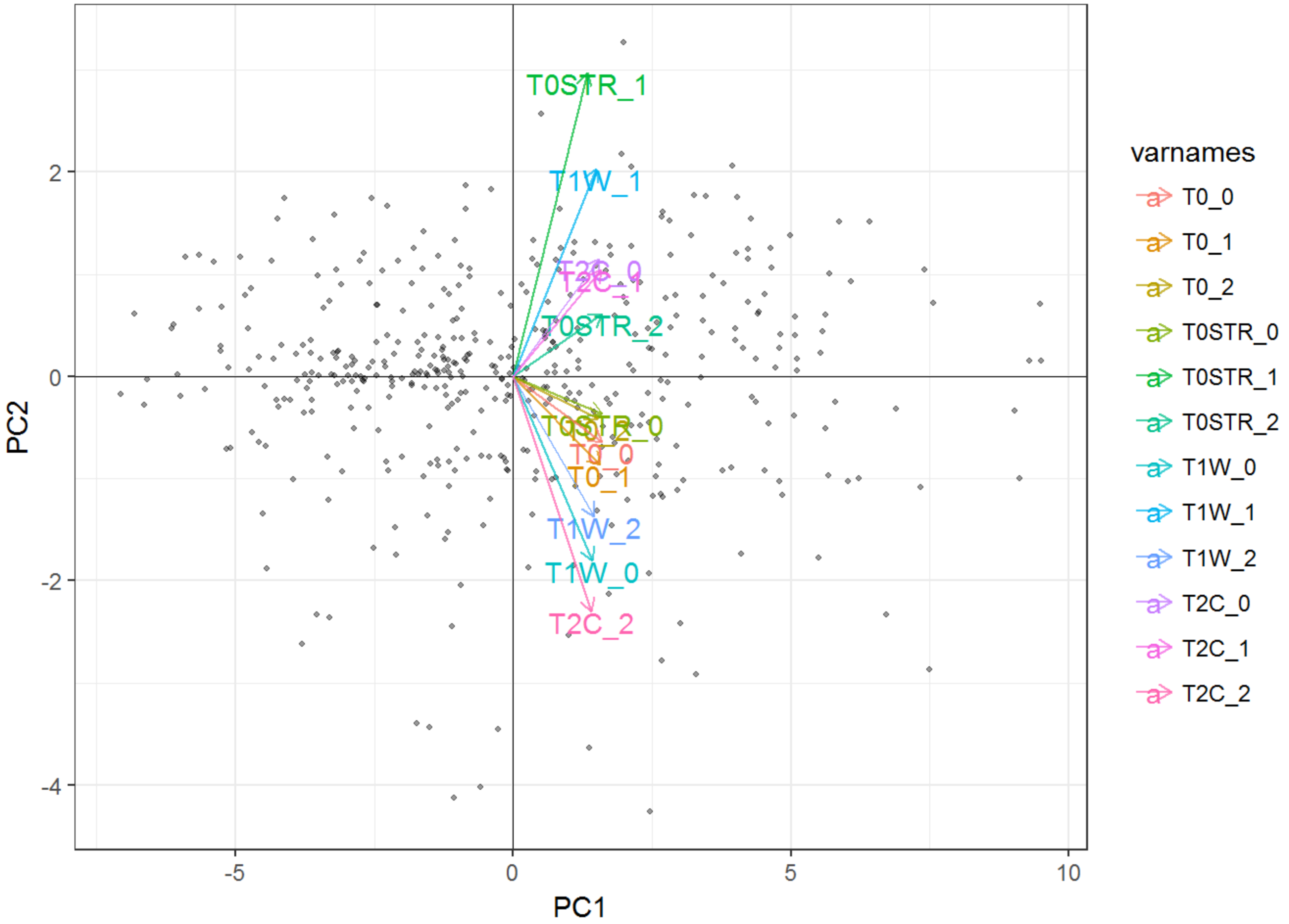

MDS plots:

```
genes.MDS <- MDSplot(genes(cuff))
genes.MDS
```

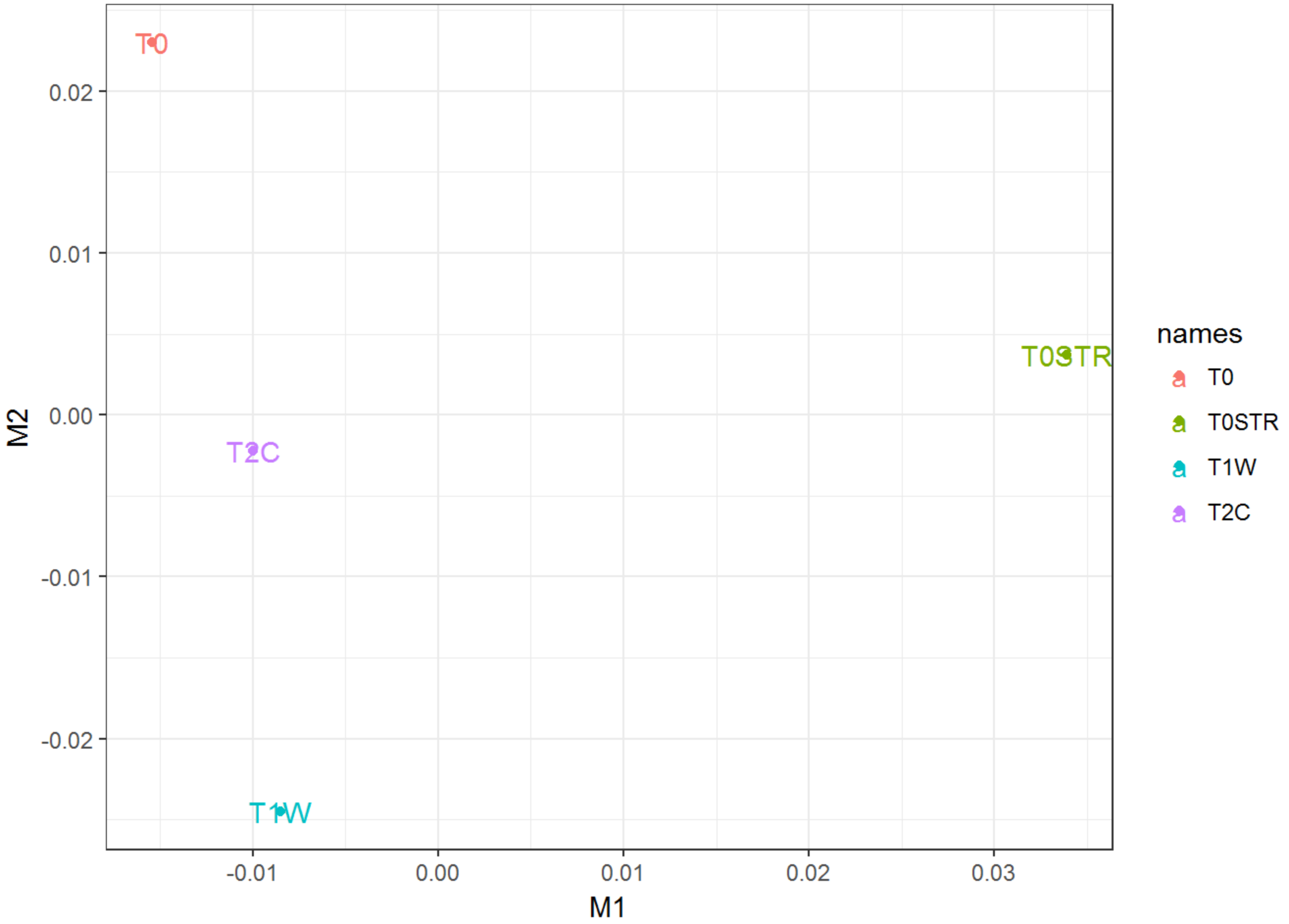


```
genes.MDS.reps <- MDSplot(genes(cuff), replicates = TRUE)
genes.MDS.reps
```

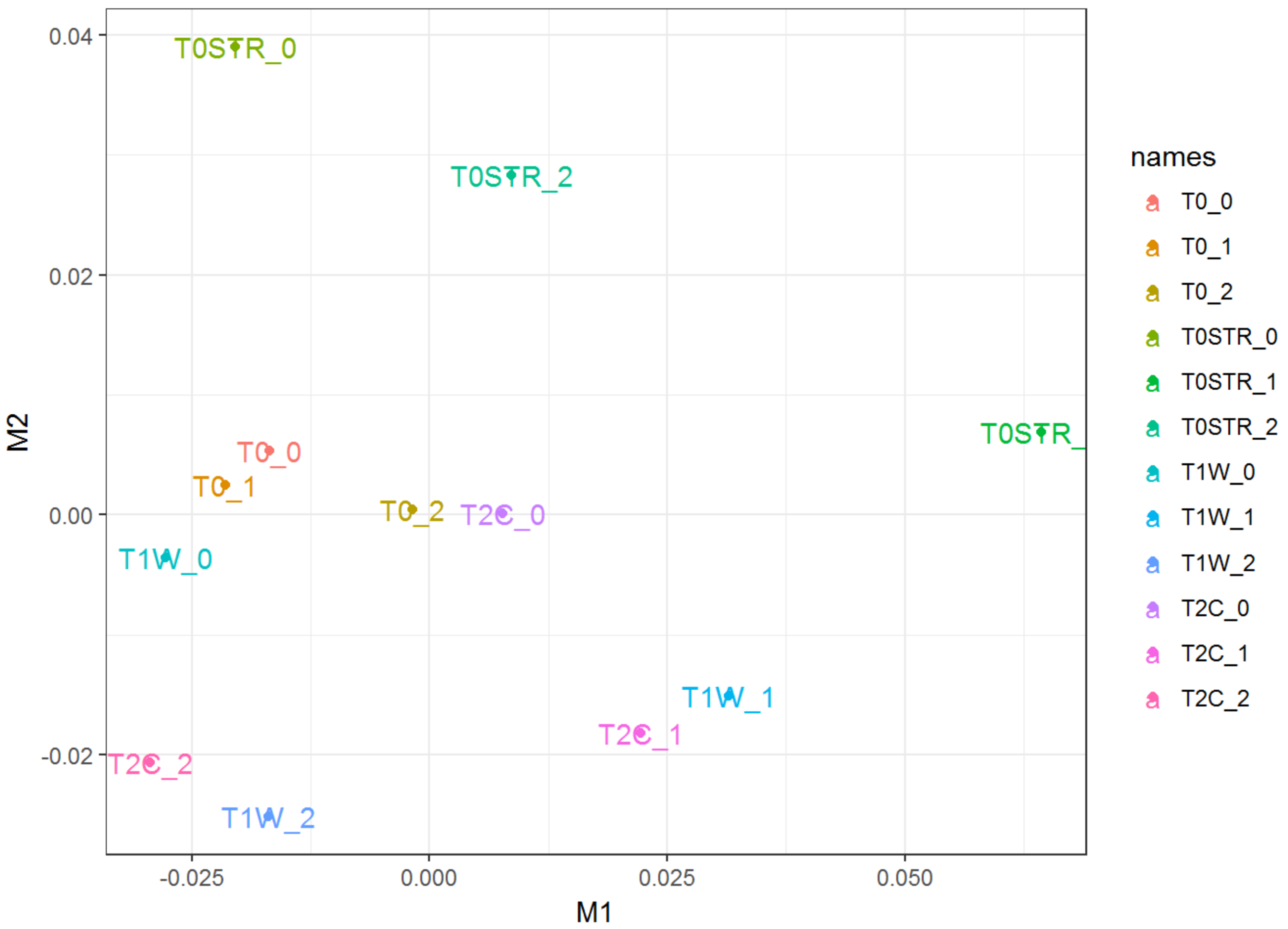


```
sig.MDS <- MDSplot(sigGenes)
```

```
## Using tracking_id, sample_name as id variables
```

```
sig.MDS
```

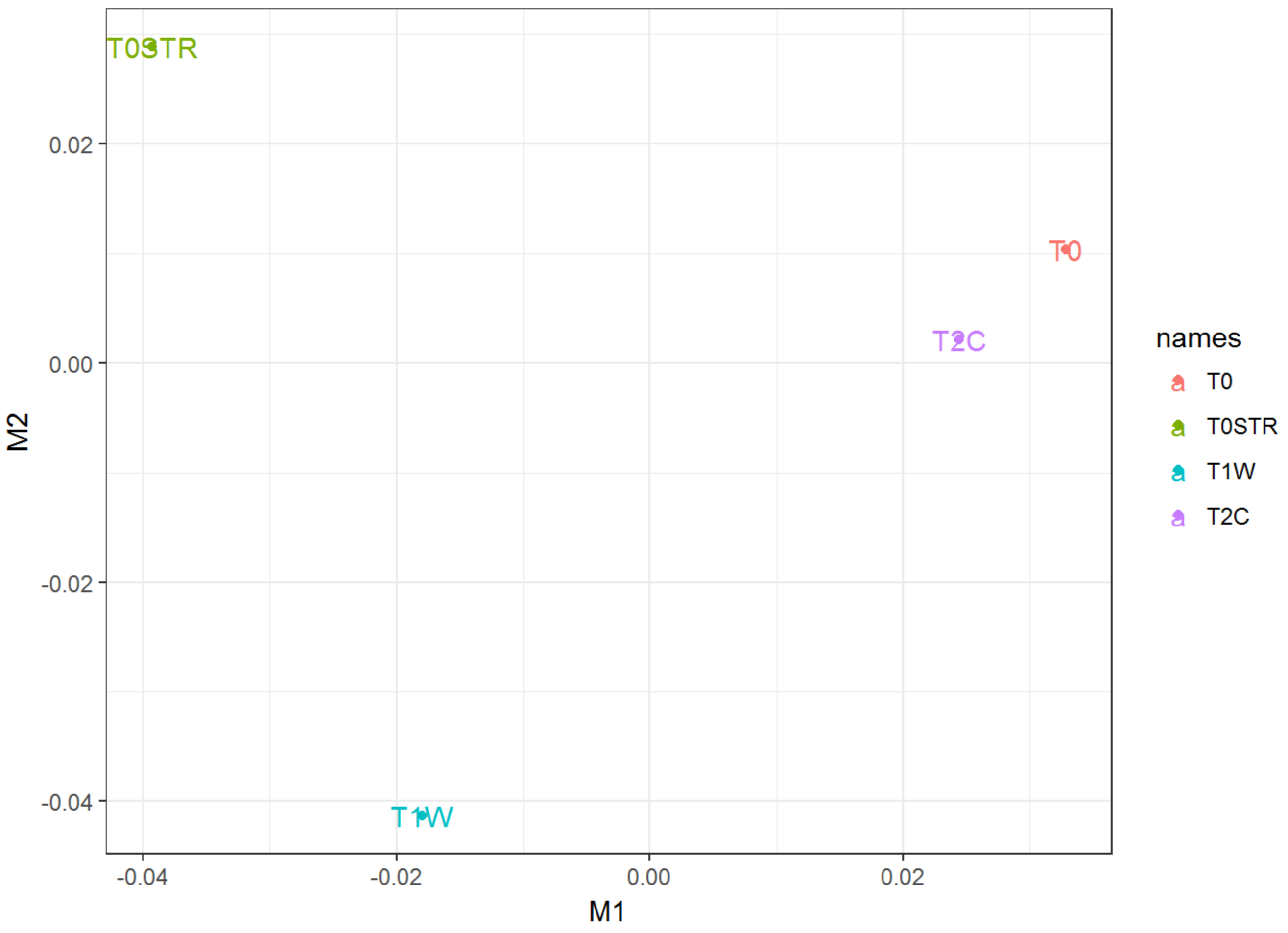


```
sig.MDS.reps <- MDSplot(sigGenes, replicates = TRUE)
```

```
## Using tracking_id, rep_name as id variables
```

```
sig.MDS.reps
```

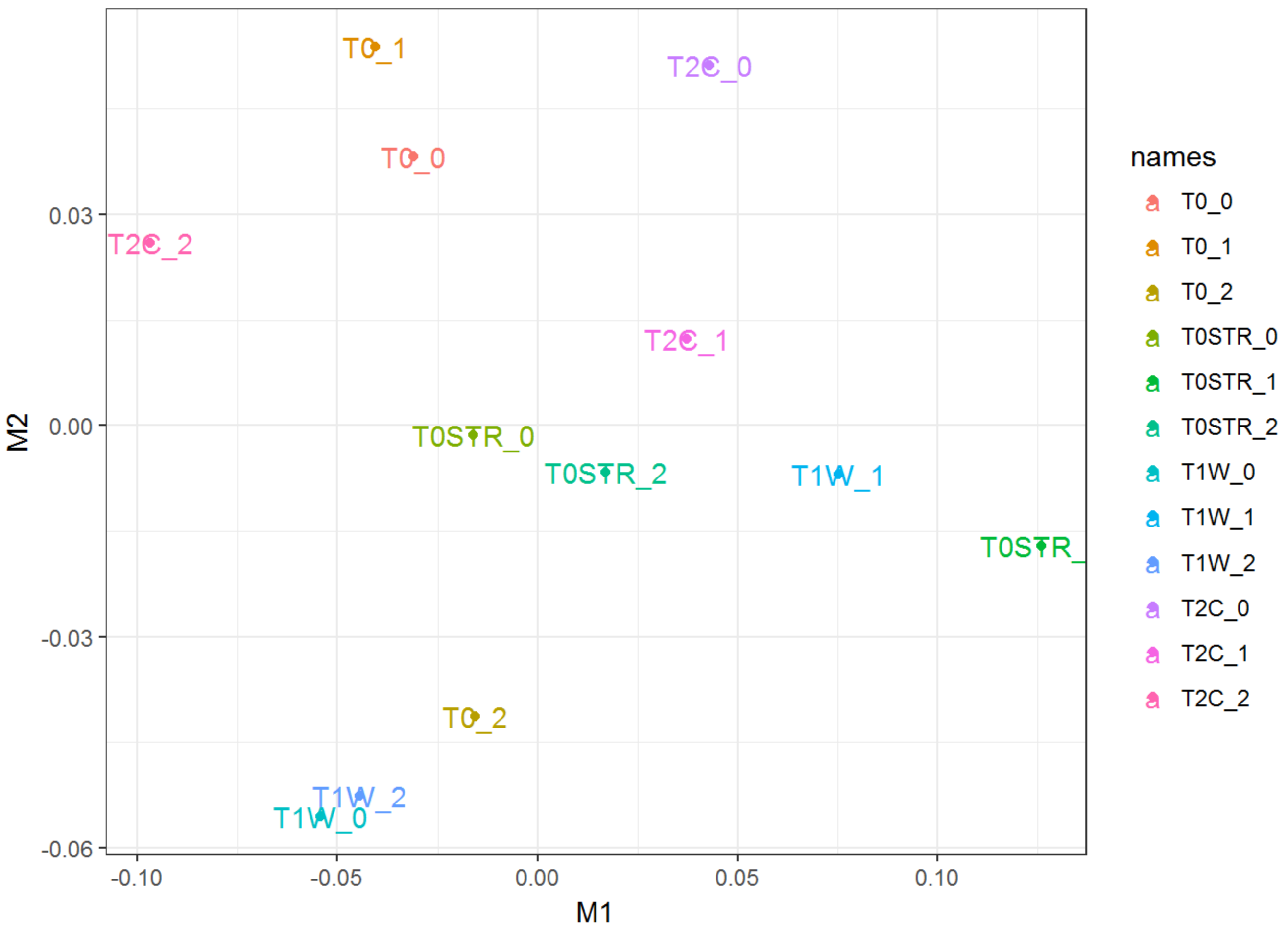

K-means clustering for significant genes:

```
ic4 <- csCluster(sigGenes, k=4)
```

```
## Loading required package: cluster
```

```
## Warning: package 'cluster' was built under R version 3.4.4
```

```
## Using tracking_id, sample_name as id variables
```

```
ic4p <- csClusterPlot(ic4)
ic4p
```

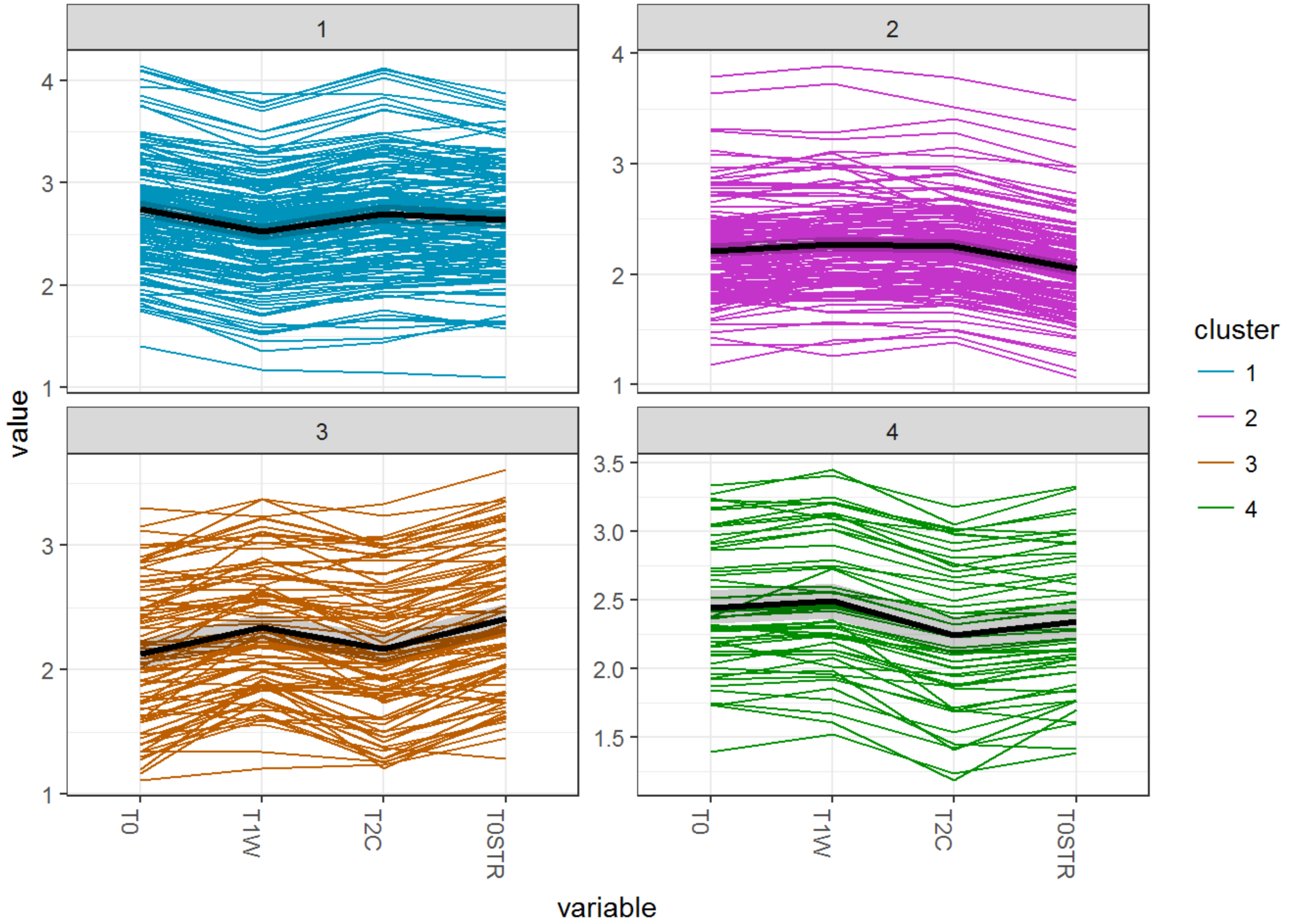


```
ic9 <- csCluster(sigGenes, k=9)
```

```
## Using tracking_id, sample_name as id variables
```

```
ic9p <- csClusterPlot(ic9)
ic9p
```

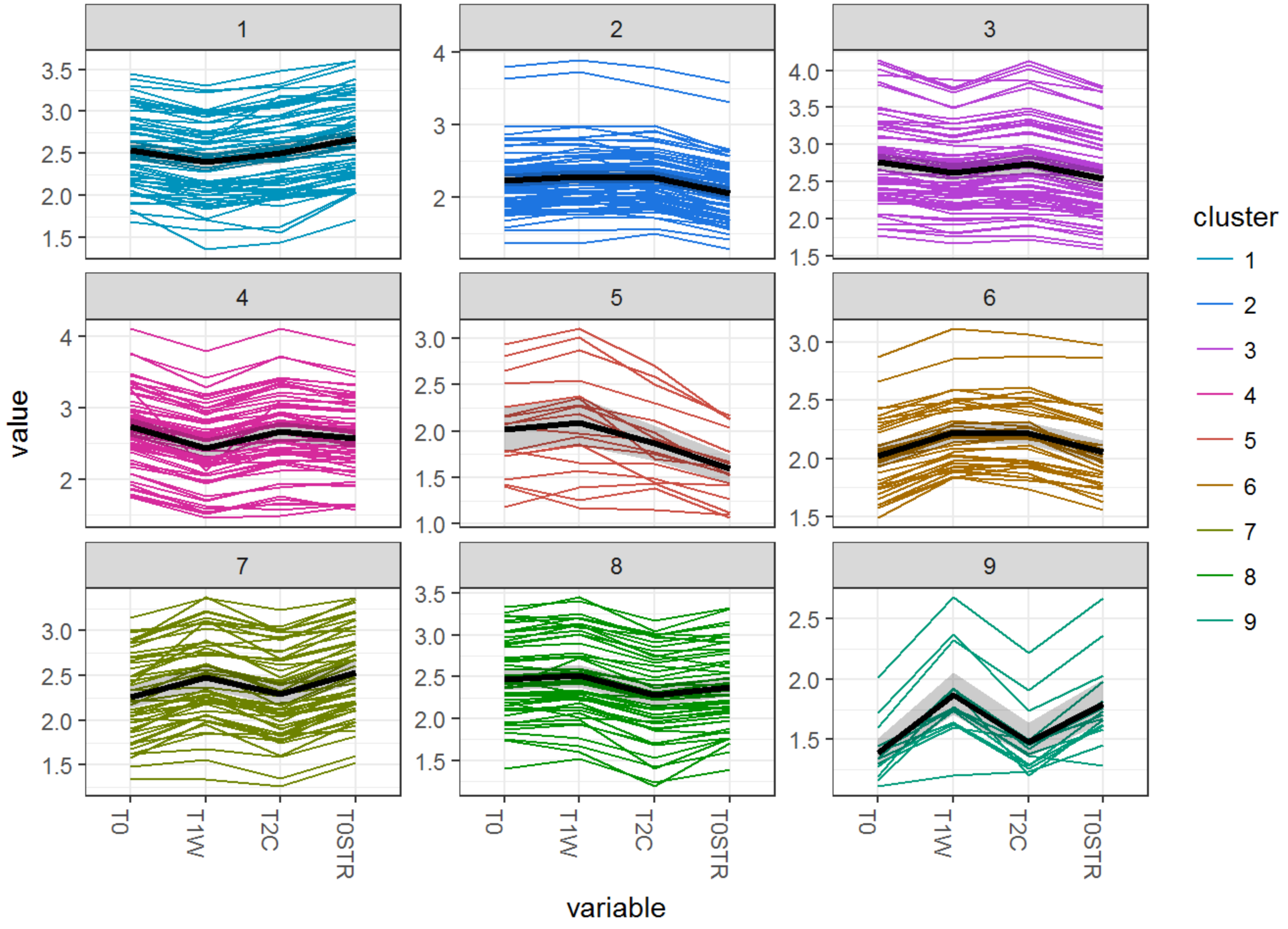


```
ic16 <- csCluster(sigGenes, k=16)
```

```
## Using tracking_id, sample_name as id variables
```

```
ic16p <- csClusterPlot(ic16)
ic16p
```

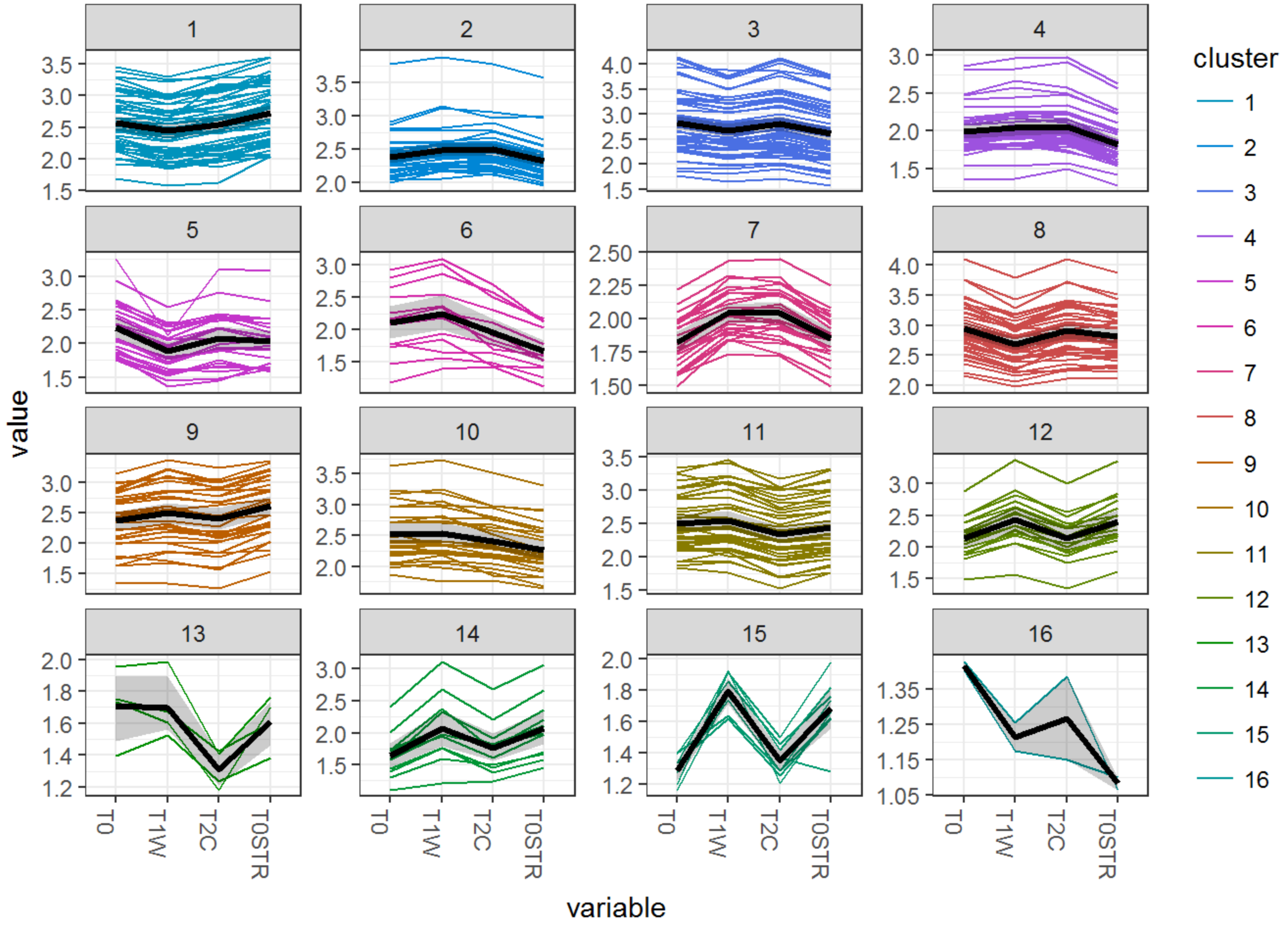


```
expressionProfile <- c(100, 10, 10, 100)
```

```
ep.plot <- expressionPlot(findSimilar(cuff, expressionProfile, n = 10))
ep.plot <- ep.plot + theme_bw()
ep.plot
```

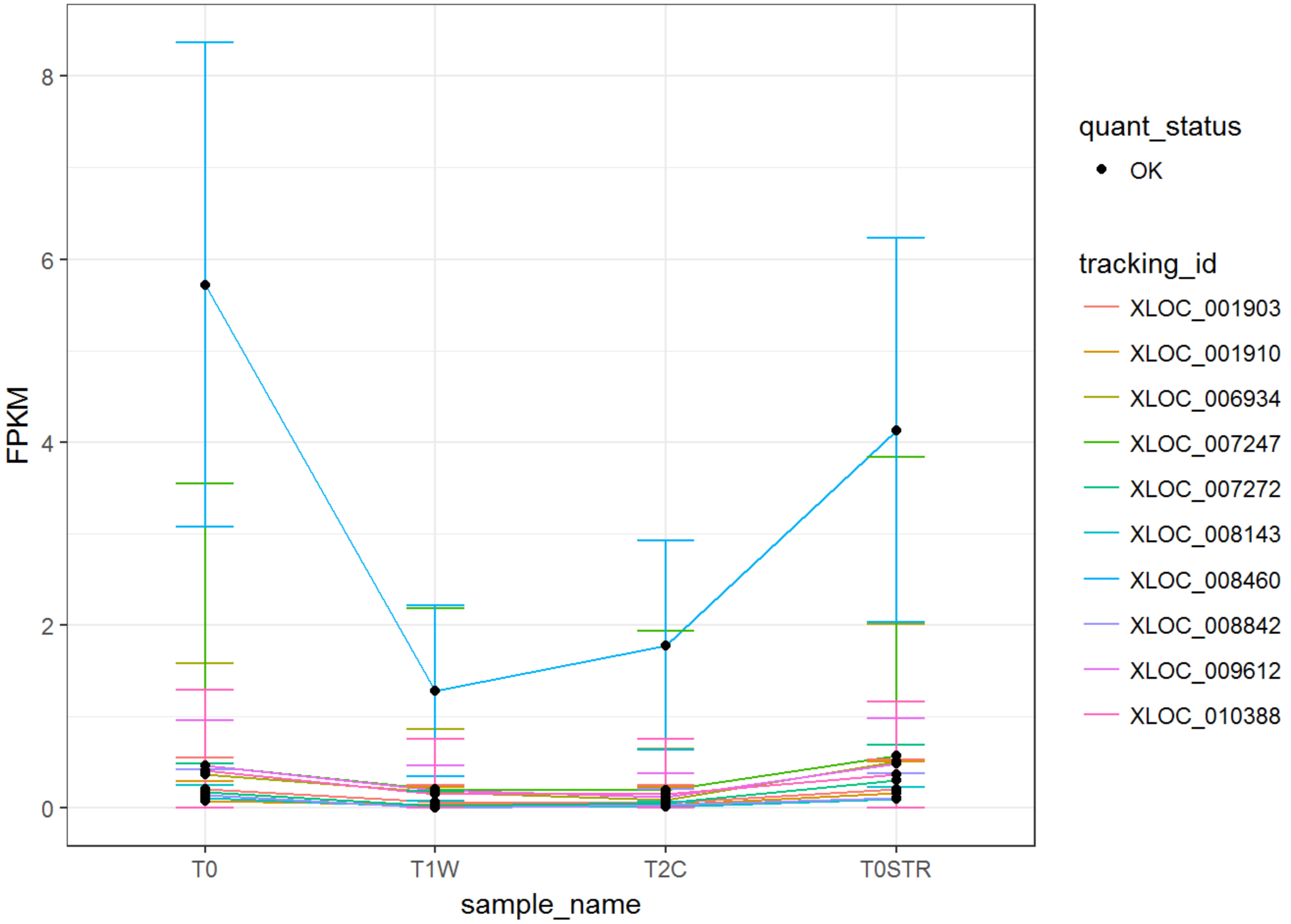
FPKM
0
2
4
6
8
T0
T1W
T2C
T0STR
sample_name
quant_status
OK
tracking_id
XLOC_001903
XLOC_001910
XLOC_006934
XLOC_007247
XLOC_007272
XLOC_008143
XLOC_008460
XLOC_008842
XLOC_009612
XLOC_010388
